# Human MicroRNA Oncogenes and Tumor Suppressors Show Significantly Different Biological Patterns: From Functions to Targets

**DOI:** 10.1371/journal.pone.0013067

**Published:** 2010-09-30

**Authors:** Dong Wang, Chengxiang Qiu, Haijun Zhang, Juan Wang, Qinghua Cui, Yuxin Yin

**Affiliations:** 1 Department of Biomedical Informatics, Peking University Health Science Center, Beijing, China; 2 Institute of Systems Biomedicine, Peking University Health Science Center, Beijing, China; 3 Department of Pathology, Peking University Health Science Center, Beijing, China; Memorial Sloan-Kettering Cancer Center, United States of America

## Abstract

MicroRNAs (miRNAs) are small noncoding RNAs which play essential roles in many important biological processes. Therefore, their dysfunction is associated with a variety of human diseases, including cancer. Increasing evidence shows that miRNAs can act as oncogenes or tumor suppressors, and although there is great interest in research into these cancer-associated miRNAs, little is known about them. In this study, we performed a comprehensive analysis of putative human miRNA oncogenes and tumor suppressors. We found that miRNA oncogenes and tumor suppressors clearly show different patterns in function, evolutionary rate, expression, chromosome distribution, molecule size, free energy, transcription factors, and targets. For example, miRNA oncogenes are located mainly in the amplified regions in human cancers, whereas miRNA tumor suppressors are located mainly in the deleted regions. miRNA oncogenes tend to cleave target mRNAs more frequently than miRNA tumor suppressors. These results indicate that these two types of cancer-associated miRNAs play different roles in cancer formation and development. Moreover, the patterns identified here can discriminate novel miRNA oncogenes and tumor suppressors with a high degree of accuracy. This study represents the first large-scale bioinformatic analysis of human miRNA oncogenes and tumor suppressors. Our findings provide help for not only understanding of miRNAs in cancer but also for the specific identification of novel miRNAs as miRNA oncogenes and tumor suppressors. In addition, the data presented in this study will be valuable for the study of both miRNAs and cancer.

## Introduction

MicroRNAs (miRNAs) are recently identified endogenous small non-coding regulatory RNAs (∼22 nt) [1], [2]. miRNA genes are transcribed as primary miRNA precursors (pri-miRNAs), which are converted into ∼100 bp precursor miRNAs (pre-miRNAs). These pre-miRNAs are further processed into ∼22 bp mature miRNAs which can exert regulatory function on target genes [3]. miRNAs normally function as negative gene regulators at the post-transcriptional level by binding to the 3′UTRs of the target mRNA through base pairing, resulting in its cleavage or translation inhibition [4], [5], [6]. Increasing evidence indicates that miRNAs play crucial roles in a variety of basic biological processes, such as metabolism, proliferation, development [7], [8], differentiation [9], apoptosis [10], [11], [12], cellular signaling [13], and immune response [14].

Emerging evidence also shows that dysfunction of miRNAs is associated with various human diseases, such as cardiovascular disease and cancer [7], [8]. It is well known that cancer is associated with very complex genetic alternations in oncogenes and tumor suppressors [7], [15]. And recent evidence shows that miRNAs also contribute to tumor formation and development, suggesting that miRNAs can function as oncogenes or tumor suppressors [7], [15], [16], [17], [18]. Moreover, these tumor associated miRNAs can serve as biomarkers for tumor diagnosis and prognosis [17] or therapeutic targets [7], [15]. Although the emergence of miRNAs as oncogenes and tumor suppressors has attracted great interest, little is known about these miRNAs. Therefore, it is of particular importance to investigate miRNA oncogenes and tumor suppressors on a large scale basis in order to improve our understanding of miRNAs and their relationship with cancer.

To accomplish this, we focus here on a group of human miRNA oncogenes (referred to as p-oncomirs) and tumor suppressors (referred to as p-mirsupps), and evaluate the function, evolutionary conservation, chromosome distribution, expression, molecule size, free energy, transcription factors, and targets of these molecules. Finally, a machine-learning based prediction model is established for use in discriminating novel human miRNA oncogenes from tumor suppressors. Our results not only present clear patterns capable of discriminating p-oncomirs from p-mirsupps, but also provide new insights into the role of miRNAs in cancer, which is of potential value in cancer diagnosis, prognosis and therapy.

## Results

### Functions of p-oncomirs and p-mirsupps

In order to summarize the functions of p-oncomirs and p-mirsupps, a miRNA function database [19] and related literature were manually culled and reviewed. As a result, the functions of p-oncomirs and p-mirsupps can be thought of as representing two basic complementary processes of cancer formation and development, namely oncogenesis and tumor-suppression (**File S1**). The p-oncomirs typically function to promote cell growth, inhibit apoptosis, repress immune cell development and control the cell cycle, while p-mirsupps function to inhibit cell growth, induce apoptosis, regulate immune cell development, and bring about cell cycle arrest. In addition, miRNA oncogenes and tumor suppressors are not invariable in function but can perform the converse function (oncogene becomes suppressor and vice versa) under specific conditions. For example, as a miRNA oncogene, mir-24 normally functions to promote lung carcinoma cell growth as reported by Cheng et al [20]. However, they also reported that inhibition of mir-24 resulted in a profound increase in Hela cell growth, representing an aspect of tumor-suppression [20].

### Evolutionary conservation of p-oncomirs and p-mirsupps

The evolutionary conservation of genes has important implications in the study of gene function, genomic organization, human disease, and medicine [21], [22]. Many miRNAs are also conserved in various species during evolution [23], [24], [25], but some miRNAs show evidence of rapid evolution [26], [27]. It has been reported that the importance of a gene is negatively correlated with its evolutionary rate [28], and as guardians of the genome, loss-function of tumor suppressors may be more critical in tumorigenesis, as demonstrated by Cui et al. [29]. However, the evolutionary patterns of p-oncomirs and p-mirsupps are largely unclear. Therefore, in order to determine the evolutionary patterns of p-oncomirs and p-mirsupps, we studied several aspects of their evolution including cross species conservation, miRNA gene duplicates, and single nucleotide polymorphisms (SNPs).

#### 1. Cross-species conservation

**Figure 1:**
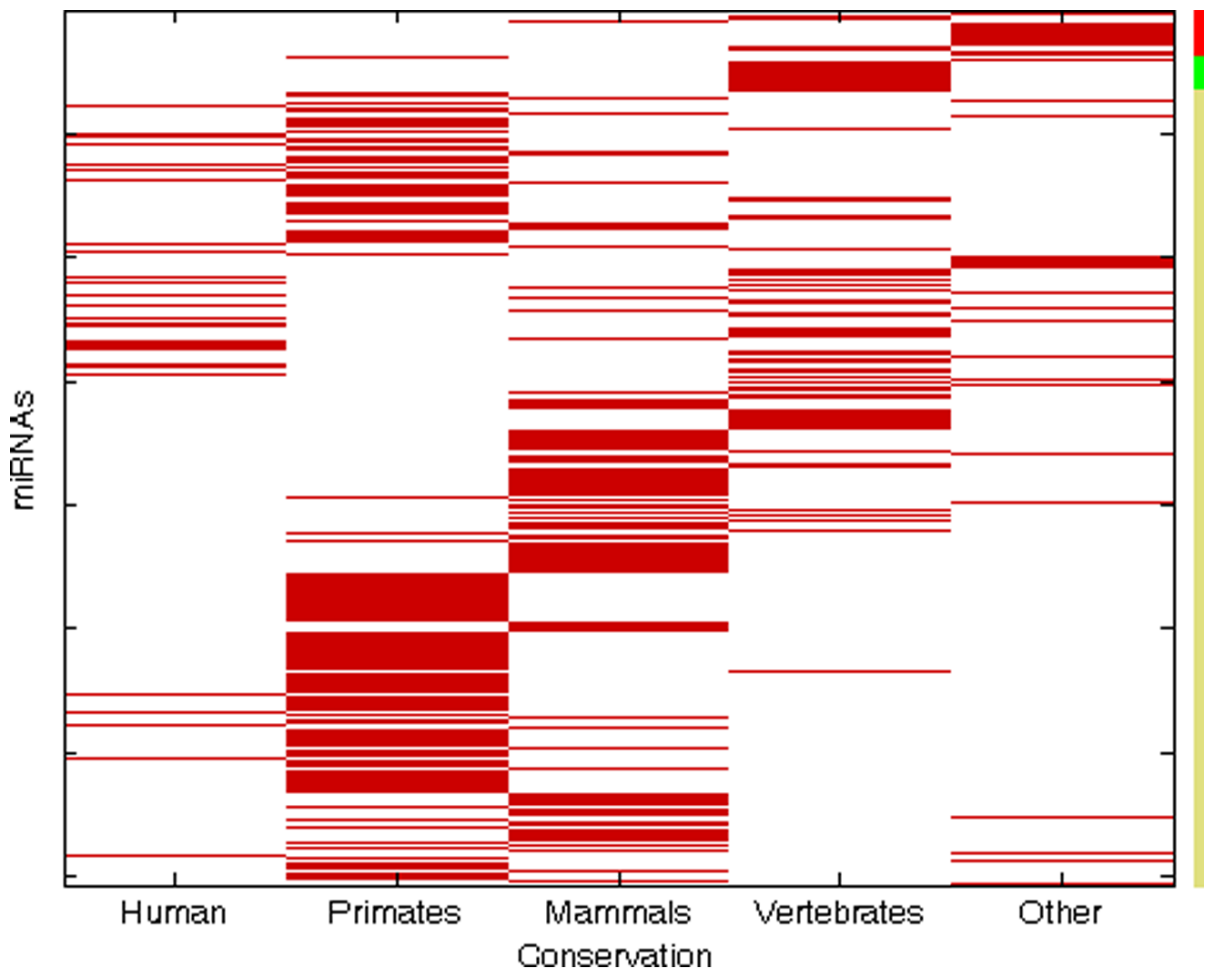
Heatmap of human miRNAs (rows) conserved across five species groups (columns). If a given miRNA is present in the corresponding conservation group, the corresponding element in the heatmap is colored as red; otherwise, this element is colored as white. The right color bar represents different miRNAs: miRNA oncogenes (red), tumor suppressors (green), and other miRNAs (yellow).

We investigated the cross-species distribution of human p-oncomirs and p-mirsupps. To do so, we classified human miRNAs into five groups based on the miRNA family data in miRBase [30] using the method of Zhang et al. (**File S2**) [26]. These groups include miRNAs that are present only in humans (G5); miRNAs that are conserved in primates (G4); conserved in mammals (G3); conserved in vertebrates (G2); or conserved in other more distant species (G1, which is the most conserved group). We found that the distribution of p-oncomirs, p-mirsupps, and other miRNAs (referred to as o-miRNAs) was markedly uneven in these five groups (**Figure 1**). The t-oncomirs (p-oncomirs and p-mirsupps referred to as tumor-associated miRNAs) were more conserved than o-miRNAs. More than 96.8% (61/63) of the t-miRNAs were present in the G1 or G2 groups, which was significantly higher than the presence of o-miRNAs (21.3%, 137/643) (P = 1.16×10^−34^, Fisher's exact test, **Figure S1a**). This result indicates that t-miRNAs may be of greater importance in molecular functions and biological processes, or conversely, that dysfunction of these molecules has a greater probability of causing cancer. In addition, p-mirsupps showed significantly higher conservation than p-oncomirs, and almost 62.2% of the p-mirsupps were conserved in the G1 group, while only 11.5% of the p-oncomirs were conserved in the G1 group (P = 5.0×10^−5^, Fisher's exact test, **Figure S1b**). This result suggests that analogous to the tumor suppressors of protein coding genes, these miRNA tumor suppressors may also be more critical in regulating tumorigenesis than miRNA oncogenes.

#### 2. Identical miRNA duplicates

Gene duplication is one of the most important mechanisms of gene function compensation, wherein, loss-of-function in one gene can be compensated by its duplicate(s) [31]. Many protein-coding genes have duplicates, and deletion of one gene in a set of duplicates is typically less critical than deletion of a singleton gene, as Gu et al. demonstrated in yeast [31]. It is therefore possible that a given gene needs duplicates as it is essential in such a way that an additional copy is required to ensure a low lethality if loss-of-function occurs in this gene [32]. Some reports have shown that duplicated genes evolve more slowly than singletons [33], which suggests that conserved (important) genes have a greater probability of having duplicates. This in turn raises the question as to whether this biologic pattern is also found among miRNAs. Some miRNA genes are distinctly different in situ but have identical mature sequences, termed as identical miRNA duplicates in this study. However, the distribution of identical miRNA duplicate in p-oncomirs and p-mirsupps is unclear, and which among the four groups, t-miRNAs, o-miRNAs, p-oncomirs, and p-mirsupps has greatest incidence of identical miRNA duplicates is unknown. Based on our analysis of miRNAs cross-species conservation, we expected that t-miRNAs would have a greater identical duplicate incidence as compared with o-miRNAs, and p-mirsupps would likely have a greater duplicate incidence than p-oncomirs. As expected, p-mirsupps have the highest duplicate incidence (28.6%, 8/28), followed by p-mirsupps (12.5%, 3/24) and o-miRNAs (9.0%, 51/567). These miRNAs thus show significantly different incidences of identical duplicates (P = 0.003, chi-square test, **Figure S1c**). The odds-ratio (OR) of t-miRNAs versus o-miRNAs is 2.35 and the OR of p-mirsupps versus p-oncomirs is 2.29. This is the first report to show that tumor-associated miRNAs tend to have a higher duplicate incidence than other miRNAs, and of it is of note that miRNA tumor suppressors tend to have a higher duplicate incidence than miRNA oncogenes.

#### 3. SNPs of p-oncomirs and p-mirsupps

Conserved genes tend to have a low frequency of SNPs, and as the most common genetic variants in the human genome [34], [35], SNPs can be used to evaluate the evolutionary conservation of miRNAs [24], [25]. It has been reported that miRNAs have a lower SNP density than their neighboring regions, consistent with the high overall conservation of miRNAs. However, as the SNP patterns of human p-mirsupps and p-oncomirs are unclear, we first mapped the SNP data (dbSNP build 129) from UCSC [36] to human miRNA precursors. Subsequent statistical analysis demonstrated that p-mirsupps, p-oncomirs, and o-miRNAs show distinct differences in SNP incidence. The p-mirsupps had the lowest SNP incidence (8.1%, 3/37), followed by the p-oncomirs (19.2%, 5/26) and the o-miRNAs (34.3%, 219/638) (P = 0.001, chi-square test, **File S3, Figure S1d**). In addition, the p-mirsupps had a lower SNP incidence (OR = 0.42) than the p-oncomirs, although the result was not significant (P = 0.19, Fisher's exact test), which may be due to the small number of SNPs in both datasets.

### Chromosome distribution of p-oncomirs and p-mirsupps

**Figure 2:**
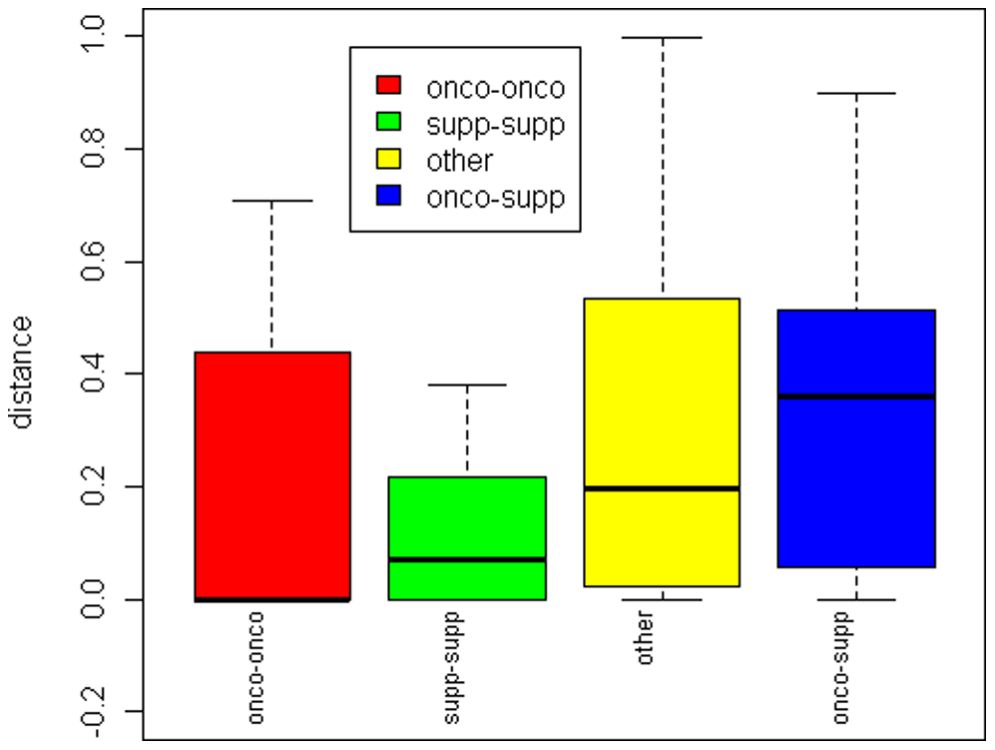
Normalized distances on chromosomes of four groups of miRNA pairs. Pairs of putative miRNA oncogenes (green), pairs of putative miRNA tumor suppressors (red), pairs of putative miRNA oncogenes and putative miRNA tumor suppressors (blue), and other pairs that don't belong to any of the first three groups (yellow).

Genes are not randomly distributed on chromosomes [22]. miRNAs tend to form clusters on chromosomes [37] and the distribution of miRNA genes is also nonrandom. In order to study the chromosome distribution of p-oncomirs and p-mirsupps, we first mapped all human miRNAs onto chromosomes (**Figure S2**). Results showed that chromosome 14 had fewer than expected t-miRNAs (P = 0.002, randomization test), and chromosome 9 (P = 0.03), 13(8/14, P = 0.0002) and 21 (P = 0.006) contained more t-miRNAs than expected. There was a weak positive correlation between the incidence of t-miRNAs and total miRNAs (R = 0.36, P = 0.09, Spearman's correlation). In addition, we also identified a positive correlation between the incidence of p-oncomirs and p-mirsupps (R = 0.45, P = 0.03, Spearman's correlation), which suggests that a high incidence of miRNA oncogenes is frequently accompanied by a high incidence of miRNA tumor suppressors. Therefore, this tendency to accompany each other at the chromosome level warranted investigation into the presence or absence of p-oncomir and p-mirsupp clustering and also raised the question as to whether they are situated closely or distantly from each other on the chromosome. To do this, we normalized the length of each chromosome as one unit and classified all pairs of miRNAs on a given chromosome into four groups: pairs consisting of p-oncomirs and p-oncomirs (referred to as “onco-onco”), pairs of p-mirsupps and p-mirsupps (referred to as “supp-supp”), pairs of p-oncomirs and p-mirsupps (referred to as “onco-supp”), and pairs that did not belong to any of these first three types of pairs (referred to as “other”). We then calculated the distances between miRNAs in all of the above pairs. It was of interest that these four groups of pairs showed significantly different distances of separation (P = 5.49×10^−7^, Kruskal-Wallis test, **Figure 2**). The p-oncomirs tended to be clustered together. The p-mirsupps also tended to be clustered together. However, the p-oncomirs and p-mirsupps tended to be separated from each other (**Figure 2**). This result indicates that at the chromosome level p-oncomirs and p-mirsupps may cluster in different regions.

Calin et al. found that over half of the human miRNAs are located in fragile sites and genomic regions involved in cancer [37]. Based on their data, we counted the number of p-oncomirs and p-mirsupps located in the deleted regions (DR) and in the amplified regions (AR) in human cancer, respectively. The results showed clear differences in distribution of p-oncomirs and p-mirsupps in these two types of regions (P = 0.001, Fisher's exact test). The p-oncomirs were more frequently found in amplified regions (8AR vs. 5DR), while the p-mirsupps were more frequently found in deleted regions (2AR vs. 21DR, **File S4**). This result indicates that in human cancer p-oncomirs often gain function, while p-mirsupps frequently lose function, which is consistent with the overall concepts of oncogenesis and tumor-suppression, respectively.

### Expression of p-oncomirs and p-mirsupps

In a manner similar to protein-coding genes, miRNAs must maintain a level of expression for proper function, and impaired expression may result in diseases [7]. Although some characteristics of miRNA expression such as tissue-specific expression [8] have been described, understanding of p-oncomir and p-mirsupp expression overall remains quite limited.

#### 1. Tissue specificity

Previous studies have shown that evolutionarily conserved protein-coding genes have a broad tissue expression profile in mammals, insects, and plants [21]. This may due to the fact that conserved genes are associated with functions which are fundamental, and operate in diverse cellular conditions, and are therefore expressed in more tissues [21]. We examined the tissue specificity of p-oncomirs and p-mirsupps in normal tissues based on the miRNA tissue specificity index (TSI) data used by Lu et al. [38]. As expected, we found that t-miRNAs are more broadly expressed than o-miRNAs (P = 1.89×10^−5^, Wilcoxon test). The p-mirsupps showed a slightly greater breadth of expression than the p-oncomirs (P = 0.26, Wilcoxon test) but this difference was not significant, and this failure to achieve significance may be due to the small sample size of both miRNAs and tissues. Tissue specificity is a relative concept as it is dependent in part on how many tissues are used in a given study. As such, a significant difference may be found in p-oncomirs and p-mirsupps when expression data for more tissues becomes available.

#### 2. Expression level

**Figure 3:**
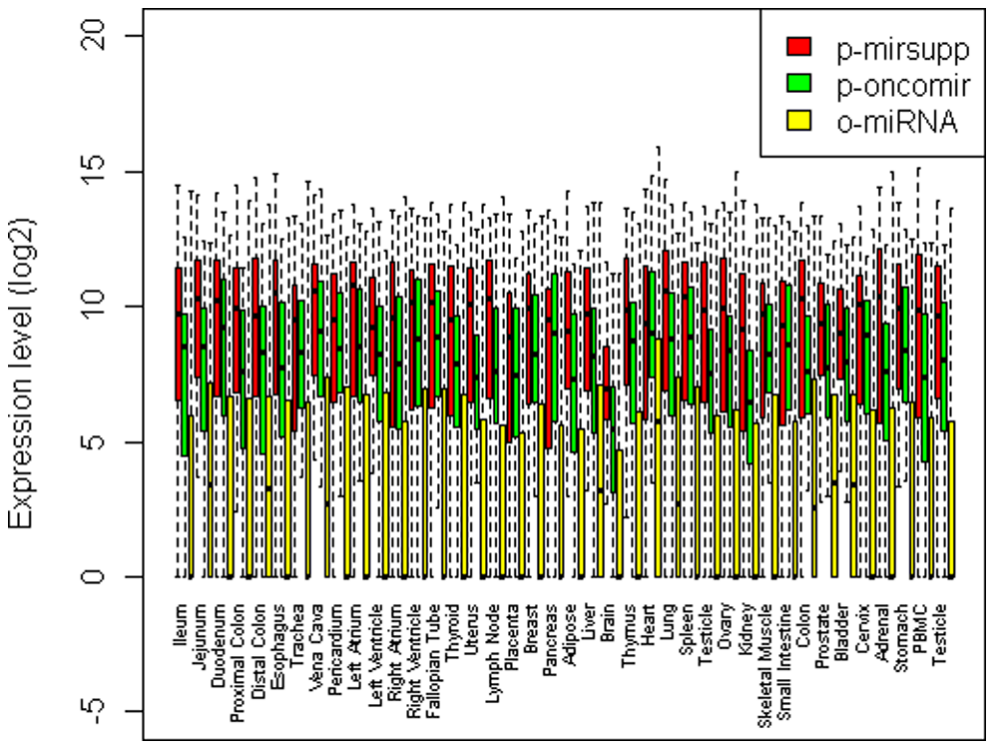
Distribution of miRNA expression levels for putative miRNA tumor suppressors (red), putative miRNA oncogenes (green), and other miRNAs (yellow) across 40 normal human tissues, shown as a box plot.

Figure 3 shows the expression level of p-oncomirs, p-mirsupps, and o-miRNAs in 40 normal human tissues based on the data of Liang et al. [39]. The p-mirsupps, p-oncomirs, and o-miRNAs show significantly different expression levels (P = 8.01×10^−11^, Kruskal-Wallis test, **Figure 3**). The p-mirsupps have the highest expression levels in almost all of the 40 tissues, followed by p-oncomirs and o-miRNAs. Considering the evolutionary conservation of p-mirsupps, p-oncomirs, and o-miRNAs as analyzed earlier, this is analogous with previous studies of protein-coding genes, wherein, expression level is correlated with evolutionary conservation [21], [40]. This observation is consistent with the concept that miRNAs with fundamental functions are required to function in more cellular conditions and more tissues, and therefore have more expression copies.

### Targets of miRNAs

Maintaining a suitable expression level is critically important for both miRNAs and protein-coding genes. The miRNAs have the capacity to “tune” the expression of a target gene to a precise level [41]. Therefore, investigation of target gene is one of the keys for understanding miRNAs. In order to ensure accuracy in this analysis, here we focused only on miRNAs with experimentally validated targets.

#### 1. Targets: tumor involvement?

**Figure 4:**
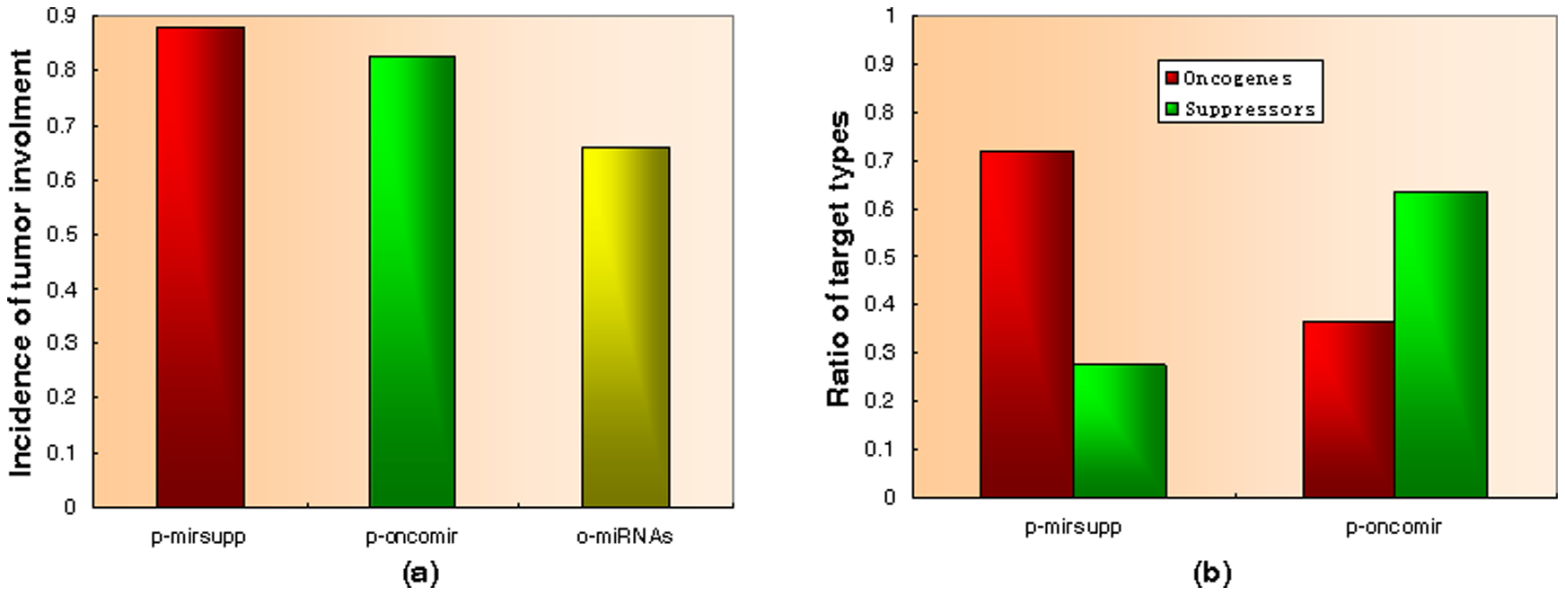
Patterns of miRNA targets. (a) Tumor involvement of targets of putative miRNA oncogenes, putative miRNA tumor suppressors, and other miRNAs. (b) Oncogene and suppressor distribution on targets of putative miRNA oncogenes and putative miRNA tumor suppressors.

It is reasonable to suppose that tumor associated miRNAs preferentially regulate tumor associated protein-coding genes, and we thus evaluated t-miRNA targets for association with tumors. Based on the listing of miRNA targets in TarBase (version 5) [42], we found that targets of p-mirsupps have the highest ratio of tumor involvement, followed by p-oncimirs and o-miRNA (P = 1.67×10^−12^, chi-square test, **Figure 4a**). Moreover, targets of t-miRNAs have a higher ratio of tumor involvement than o-miRNAs (P = 3.8×10^−13^, Fisher's exact test) and targets of p-mirsupps have a higher ratio of tumor involvement than p-oncomirs (P = 0.03, Fisher's exact test).

#### 2. Targets: oncogenes or tumor suppressors?

Zhang et al. speculated that miRNA oncogenes/tumor suppressors may exert their function in cancer pathogenesis by regulating tumor suppressors/oncogenes [15], but this could not be confirmed due to the limited number of experimentally validated target at the time of that report. Their hypothesis is supported by individual evidence [18], however, no statistical analysis has been available. Therefore, we performed an analysis of target oncogenes and tumor suppressors of p-oncomirs and p-mirsupps based on the data in TarBase [42]. As anticipated, p-oncomirs and p-mirsupps showed clear differences, with p-oncomirs preferentially targeting tumor suppressors, while p-mirsupps target oncogenes (P = 0.025, Fisher's exact test, **Figure 4b**). This result gives statistical confirmation to the speculation that tumor associated miRNAs contribute to tumorigenesis by functional interactions with oncogenes and tumor suppressors [18].

#### 3. Targets: mechanisms of action

miRNAs typically negatively regulate gene expression. Although the exact mechanisms through which miRNAs regulate gene expression remains under debate, a simplified view of this regulation is that miRNAs can inhibit translation or induce cleavage of target mRNAs. Precise complementarity results in cleavage of target mRNAs, while the binding of miRNAs and mRNAs with mismatches leads to translation inhibition of target mRNAs [8], [43]. In animals, the cleavage of mRNAs by miRNAs occasionally occurs but is much more unusual [43]. How miRNAs oncogenes and tumor suppressors function remains unknown, but understanding the mechanisms through which miRNAs contribute to tumorigenesis is of clear importance. Here, we classified miRNA-target actions into a cleavage group and an inhibition group. Interestingly, p-oncomirs, p-mirsupps, and o-miRNAs showed significantly different distribution in these two groups (P = 0.0004, chi-square test, **Figure S3**). There was a significantly higher percentage (42.1%) of tumor-associated miRNAs (t-miRNAs) in the cleavage group than o-miRNAs (21.4%) (P = 0.002, Fisher's exact test). Of even greater interest, over half (53.3%) of the actions between p-mirsupps and their targets are of cleavage type, which is significantly higher than that of p-oncomirs and their targets (25.8%) (P = 0.01, Fisher's exact test). Further experiments are needed to determine why t-miRNAs, and especially p-mirsupps, tend to act by cleavage mechanism, but we speculate that one possible reason may be that translation inhibition confers potential danger for target mRNA re-activation, whereas cleavage ensures target inactivation. Although further experiments are needed to explore why tumor-associated miRNAs, especially the miRNA tumor suppressors, more frequently act by cleavage on their targets, this study highlights the fact that different modes of action exist among miRNAs and opens a door for studying the exact mechanisms by which they contribute to tumorigenesis.

### Size, free energy, transcription factors, and prediction model of p-oncomirs and p-mirsupps

In this study, we also analyzed the molecular size, free energy, and transcription factors of miRNA oncogenes and tumor suppressors (**File S5**). In addition, a prediction model of p-oncomirs and p-mirsupps was also established (**File S5**).

## Discussion

This study not only identified putative human miRNA oncogenes and tumor suppressors on a large scale but also greatly expanded current knowledge about miRNAs and cancer. Features of miRNA oncogenes and tumor suppressors which were analyzed included function, evolutionary conservation, chromosomal distribution, molecular size, free energy, transcription factor, and targets. We found that miRNA oncogenes and tumor suppressors show different patterns in most of the above features.

Perhaps of greatest importance, the patterns identified in this study allow accurate classification of newly identified miRNAs as p-oncomirs and p-mirsupps which function primarily in oncogenesis and tumor-suppression respectively. It is noteworthy that miRNA oncogenes and tumor suppressors are not invariable in their function but can switch function in some circumstance. However, the model established in this study with the miRNA data from before 2008 was tested with the data from after 2008 and found to be consistent in classifying t-miRNAs. Nonetheless our current knowledge of these molecules is in many ways still rudimentary, however, if more data regarding novel cancer gene mutations, genome methylation status and so on are integrated into future studies, it is likely that our understanding of miRNA-cancer associations will continue to grow.

Finally, we hope the data presented in this study will be a resource for a wide range of research exploring both miRNAs and cancer as many important issues remain to be considered in future studies. One important issue is whether the role played by miRNAs differs in different types of cancer. A second question is that although it is clear that oncogenic miRNAs may act as tumor suppressors and vice versa, when, where, and how do such interchanges of role occur. Although it is not easy to answer such questions, it is likely that their resolution will ultimately provide great help in understanding the role of miRNAs in cancer. It is also useful to note that the experimental confirmation of these observations is of critical importance to determine how miRNAs contribute to tumorigenesis. For example, regarding the mechanism of action of miRNA oncogenes and suppressors, it is unclear why the miRNA suppressors tend to abolish their target mRNAs directly but not just inhibit their translation; and how the miRNA suppressors achieve this.

### Conclusion

In conclusion, although we are only beginning to understand miRNA oncogenes and tumor suppressors in cancer, here we have found significantly different patterns in miRNA oncogenes and tumor suppressors. This study represents the first large-scale analysis of human miRNA oncogenes and miRNA tumor suppressors. The patterns identified in this study will provide help in understanding of the roles miRNAs play in cancer, in the identification of biomarkers for cancer diagnosis, prognosis, and therapy, in the identification of novel miRNA oncogenes and tumor suppressors, and in guiding biologic experimentation.

## Materials and Methods

### Identifying the human p-oncomirs and p-mirsupps

We identified human miRNAs either as p-oncomir or as p-mirsupp by manually reviewing and culling nearly 5,000 publications. The decision rules for separating p-oncomirs and p-mirsupp were as follows. If one (or more) paper clearly suggested a given miRNA was a putative oncogene or tumor suppressor, we assigned it to the p-oncomir or p-mirsupp group, respectively. If no paper clearly suggested that a given miRNA was a putative oncogene or tumor suppressor, it was designated as a p-oncomir only if at least three different papers provided strong evidence that the molecule in question was associated with up-regulation, overexpression, and/or tumor growth; or designated as a p-mirsupp if associated with down-regulation, underexpression, and/or inhibition of tumor growth. In addition, assignments into these two groups also required that no conflicts about the given miRNA were found in literature. A few miRNAs were found which evidently act as either oncogenes or tumor suppressors depending on the context [44], and these were not assigned into either the p-oncomir or p-mirsupp group. As a result, 84 miRNAs were identified as putative oncogenes or tumor suppressors (**File S1**). We further segregated these miRNAs into two groups. The miRNAs identified from papers published before June 2008 comprised the first group, and this group was used to analyze the p-oncomirs and p-mirsupps in this study. The miRNAs identified from papers published between June 2008 and April 2009 comprised the second group, which was used as the testing dataset for the miRNA oncogene and tumor suppressor prediction model.

It was important to determine how the miRNAs we identified in this study cover the total set of human miRNA oncogenes and tumor suppressors. Although it is difficult to estimate the coverage of these miRNAs to the total number of miRNA oncogenes and tumor suppressors, a simple estimation was made by comparing miRNAs with protein-coding genes. Currently, ∼700 miRNA genes have been reported in humans (miRBase, May 2009) [30] and it is estimated that there are ∼1,000 miRNAs in the human genome [7], and the p-oncomirs and p-mirsupps represent 8.4% of the human miRNAs. According to a previous estimate based on the tumor associated gene database (http://www.binfo.ncku.edu.tw/TAG/), among the total ∼30,000 protein-coding genes in the human genome [45], only 1.24% (372) are oncogenes and tumor suppressors. However, according to a much larger dataset presented by Cui et al. [29], 7.1% (2128) of human protein-coding genes are tumor associated. This percentage is similar to that of the miRNAs (8.4%). Although it is difficult to make a more specific evaluation of the coverage of the identified miRNA oncogenes and tumor suppressors to the real total number of miRNA oncogenes and tumor suppressors, this comparison is a rough estimate that suggests our data is representative and can be used for further analysis.

### Data of miRNA targets, TFs, expression, deleted region and amplified region involved in human cancers

To ensure the accuracy of this study, most of the data used in this study have experimental supporting. We obtained the experimentally supported miRNA targets from TarBase (version 5) [42], which also provides the data whether a target is involved in tumor function and provides the data of miRNA-target regulatory type (cleavage or repression). We obtained the human TF-miRNA regulatory data from TransmiR (http://cmbi.bjmu.edu.cn/transmir) [46], a database that integrated the experimentally supported TF-miRNA regulatory data, which also provides that data of TF action type (activation or repression) as well. We obtained miRNA expression data across 40 normal human tissues from Liang et al.’ study [39] and obtained the calculated miRNA tissue specificity index value based on these data from Lu et al.’ study [38]. The deleted region and amplified region involved in human cancers were obtained from Calin et al.’ study [37].

### Reliability of evaluation of putative miRNA oncogenes and tumor suppressors used in the analysis

In order to evaluate the reliability and accuracy of the categorization of the putative miRNA oncogenes and tumor suppressors used in the analysis. Five aspects of the categorization of p-oncomirs and p-suppmirs culled in this study were evaluated.

#### 1. Genomic regions of p-oncomirs and p-suppmirs

As demonstrated previously, we found that p-oncomirs and p-suppmirs are located mainly in the amplified regions and deleted regions respectively in human cancers (P = 0.001, Fisher's exact test), which suggests that the miRNAs we examined are indeed expressed in the oncogenic regions and tumor-suppressing regions.

#### 2. Targets of the p-oncomirs and the p-suppmirs

In the previous sections, we showed that p-oncomirs mainly regulate tumor suppressors, and p-suppmirs mainly regulate oncogenes (P = 0.025, Fisher's exact test). This result was consistent with the hypothesis that miRNA oncogenes and tumor suppressors preferentially interact with tumor suppressors and oncogenes, respectively, and further supported categorization of the p-oncomirs and p-mirsupps used for this study.

#### 3. Transcription factors that regulate p-oncomirs and p-mirsupps

This high percentage of balanced TF-miRNA (85.8%) regulations (**File S5**) represents qualitative evidence that the p-oncomirs and p-mirsupps are accurately assigned into these groups.

#### 4. Further review of the literature regarding the p-oncomirs and the p-mirsupps used in this analysis

In this study, for initial analysis we used p-oncomirs and p-mirsupps culled from literature published before June 2008. We later confirmed the roles of these miRNAs using literature published from June 2008 and April 2009. We found that 93.7% (59/63) of the functional descriptions of these culled miRNAs were consistently maintained over both periods. The four exceptions were mir-16-1, mir-17, mir-181b-1, and mir-181b-2. As a putative miRNA oncogene, most of the new literature supports up-regulation of miR-17-5p in cancers but Cloonan et al. reported that it may act as a suppressor in some situations [47]. As a putative miRNA tumor suppressor, miR-16 was reported to be up-regulated in malignant renal cell cancer compared to a non-malignant kidney tumor and was also up-regulated in cervical cancer [48]. miR-181b was reported to show increased expression in early onset colorectal carcinoma [49]. Considering the fact that the oncogenic and the tumor-suppressing process are interchangeable in specific cases [29], the above exceptions are reasonable and acceptable. Thus, although there were some exceptions exist, the categorization of the majority (93.7%) of these miRNAs has remained unchanged.

#### 5. Prediction of novel p-oncomirs and p-mirsupps

Most importantly, if these p-oncomirs and p-mirsupps are representative, the patterns identified based on these miRNAs should have strong predictive value for categorization of novel p-oncomirs and p-mirsupps. In fact, our prediction model achieved an accuracy of 85.7% in discrimination of novel p-oncomirs from p-mirsupps (**File S1 & File S5**). This result suggests that the p-oncomirs and p-mirsupps we culled from earlier literature are representative of oncogenes and suppressors. In conclusion, based on the above evaluations of these five aspects of p-oncomirs and p-mirsupps, we conclude that the p-oncomirs and the p-mirsupps used for this analysis were categorized correctly.

### Statistical analysis

Statistical computing and test were carried out using R, a statistical computing language (http://www.r-project.org/). We performed randomization tests to evaluate the statistical significance of enrichment of tumor-associated miRNAs on chromosomes by randomly permuting the distributions of tumor-associated miRNAs on chromosomes 5,000 times and comparing the random number of tumor-associated miRNAs on a given chromosome with the real number of tumor-associated miRNAs on this chromosome.

## Supporting Information

Figure S1 Conservation evaluation of miRNAs. (a) Comparison of cross-species conservation between tumor-associated miRNA and other miRNAs. (b) Comparison of cross-species conservation between putative miRNA oncogenes and miRNA tumor suppressors. (c) Incidences of miRNA duplicates in putative miRNA oncogenes, miRNA tumor suppressors, and other miRNAs. (d) Incidences of SNPs in putative miRNA oncogenes, miRNA tumor suppressors, and other miRNAs.
(1.53 MB TIF)

Figure S2 Chromosome distribution of putative miRNA oncogenes, miRNA tumor suppressors and other miRNAs. Each gray vertical bar represents one chromosome. MiRNAs in chromosomes are highlighted as colored lines, putative miRNA oncogenes (green), miRNA tumor suppressors (red), and other miRNAs (yellow).
(0.93 MB TIF)

Figure S3 Incidence of cleavage action on targets of putative miRNA oncogenes, putative miRNA tumor suppressors, and other miRNAs.
(0.37 MB TIF)

File S1 Information of miRNA oncogenes and tumor suppressors used in this study.
(0.26 MB DOC)

File S2 Cross-species conservation of human miRNAs.
(0.69 MB DOC)

File S3 Distribution of SNPs in human miRNAs.
(0.23 MB DOC)

File S4 Distribution of miRNA oncogenes and tumor suppressors on chromosome regions associated with human cancer. "D" represents deleted regions in cancer and "A" represents amplified regions in cancer.
(0.05 MB DOC)

File S5 Supplementary results for "Human MicroRNA Oncogenes and Tumor Suppressors Show Significantly Different Biological Patterns: From Functions to Targets."
(0.06 MB DOC)
