# Supplementary figures and images for: Human MicroRNA Oncogenes and Tumor Suppressors Show Significantly Different Biological Patterns: From Functions to Targets
Source: PLoS One. 2010 Sep 30;5(9):e13067. doi: 10.1371/journal.pone.0013067 (PMC2948010; doi:10.1371/journal.pone.0013067)

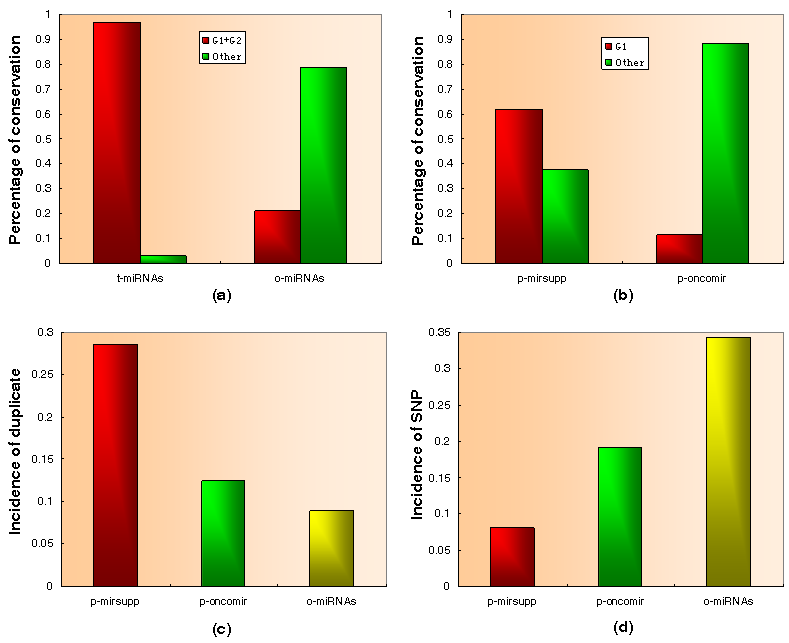

Supplement: Figure S1 — Conservation evaluation of miRNAs. (a) Comparison of cross-species conservation between tumor-associated miRNA and other miRNAs. (b) Comparison of cross-species conservation between putative miRNA oncogenes and miRNA tumor suppressors. (c) Incidences of miRNA duplicates in putative miRNA oncogenes, miRNA tumor suppressors, and other miRNAs. (d) Incidences of SNPs in putative miRNA oncogenes, miRNA tumor suppressors, and other miRNAs. (1.53 MB TIF) [file pone.0013067.s001.tif]

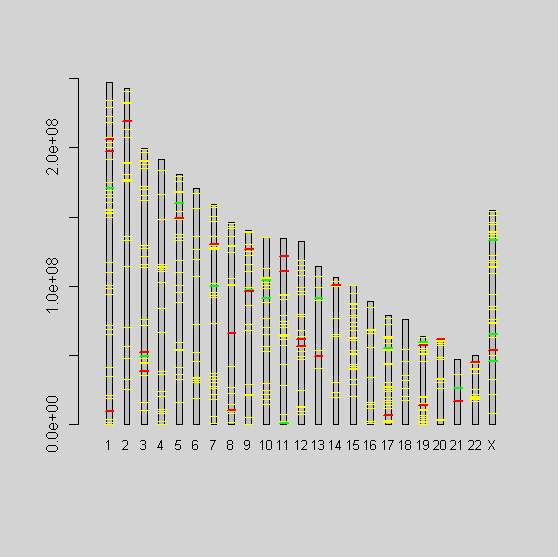

Supplement: Figure S2 — Chromosome distribution of putative miRNA oncogenes, miRNA tumor suppressors and other miRNAs. Each gray vertical bar represents one chromosome. MiRNAs in chromosomes are highlighted as colored lines, putative miRNA oncogenes (green), miRNA tumor suppressors (red), and other miRNAs (yellow). (0.93 MB TIF) [file pone.0013067.s002.tif]

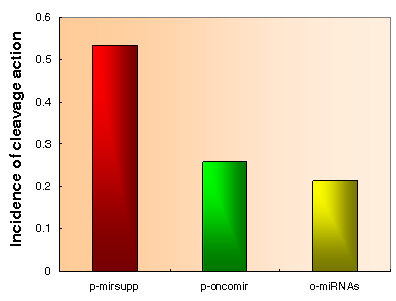

Supplement: Figure S3 — Incidence of cleavage action on targets of putative miRNA oncogenes, putative miRNA tumor suppressors, and other miRNAs. (0.37 MB TIF) [file pone.0013067.s003.tif]
