# Supplementary material for: Human MicroRNA Oncogenes and Tumor Suppressors Show Significantly Different Biological Patterns: From Functions to Targets
Source: PLoS One. 2010 Sep 30;5(9):e13067. doi: 10.1371/journal.pone.0013067 (PMC2948010; doi:10.1371/journal.pone.0013067)
Supplement: File S1 — Information of miRNA oncogenes and tumor suppressors used in this study. (0.26 MB DOC) [file pone.0013067.s004.doc]

**Supplementary File 1. Information of miRNA oncogenes and tumor suppressors used in this study.**

**Table 1. microRNA oncogenes and tumor suppressors used in the analysis dataset and their functions**

| miRNA | miRNA Type | Function |
| --- | --- | --- |
| hsa-mir-106a | ONCO | oncogenic; |
| hsa-mir-106b | ONCO | play a key role in the development of TGFbeta resistance in gastric cancer; induced cell cycle |
| hsa-mir-107 | ONCO | na |
| hsa-mir-146a | ONCO | NF-kappaB-dependent induction of microRNA miR-146, an inhibitor targeted to signaling proteins of innate immune responses |
| hsa-mir-146b | ONCO | NF-kappaB-dependent induction of microRNA miR-146, an inhibitor targeted to signaling proteins of innate immune responses |
| hsa-mir-155 | ONCO | miR-155 is activated upon B-cell receptor triggering during B cells differentiation; contribution of specific miRNA miR-126 to vascular inflammation and diseases |
| hsa-mir-17 | ONCO | accelerated the development of a B cell lymphoma;enhances lung cancer cell growth;promotes the angiogenesis;accelerates adipocyte differentiation;inhibits B cell development |
| hsa-mir-18a | ONCO | accelerated the development of a B cell lymphoma;enhances lung cancer cell growth;promotes the angiogenesis;accelerates adipocyte differentiation;inhibits B cell development |
| hsa-mir-191 | ONCO | na |
| hsa-mir-19a | ONCO | accelerated the development of a B cell lymphoma;enhances lung cancer cell growth;promotes the angiogenesis;accelerates adipocyte differentiation;inhibits B cell development |
| hsa-mir-19b-1 | ONCO | accelerated the development of a B cell lymphoma;enhances lung cancer cell growth;promotes the angiogenesis;accelerates adipocyte differentiation;inhibits B cell development |
| hsa-mir-19b-2 | ONCO | accelerated the development of a B cell lymphoma;enhances lung cancer cell growth;promotes the angiogenesis;accelerates adipocyte differentiation;inhibits B cell development |
| hsa-mir-20a | ONCO | accelerated the development of a B cell lymphoma;enhances lung cancer cell growth;promotes the angiogenesis;accelerates adipocyte differentiation;inhibits B cell development |
| hsa-mir-21 | ONCO | inhibits the apoptosis;increases cells anchorage-independent growth;promotes cancer cell growth; |
| hsa-mir-210 | ONCO | Modulates Endothelial Cell Response to Hypoxia and Inhibits the Receptor Tyrosine Kinase Ligand Ephrin-A3 |
| hsa-mir-214 | ONCO | induces cell survival; |
| hsa-mir-221 | ONCO | promotes cancerous growth; inhibits the angiogenesis; |
| hsa-mir-222 | ONCO | promotes cancerous growth; inhibits the angiogenesis; |
| hsa-mir-223 | ONCO | involved in osteoclast differentiation;restores cell differentiation |
| hsa-mir-24-1 | ONCO | promotes skeletal muscle differentiation; ;promotes cancer cell growth |
| hsa-mir-24-2 | ONCO | promotes skeletal muscle differentiation; ;promotes cancer cell growth |
| hsa-mir-372 | ONCO | oncogene |
| hsa-mir-373 | ONCO | oncogene |
| hsa-mir-92a-1 | ONCO | accelerated the development of a B cell lymphoma;enhances lung cancer cell growth;promotes the angiogenesis;accelerates adipocyte differentiation;inhibits B cell development |
| hsa-mir-92a-2 | ONCO | accelerated the development of a B cell lymphoma;enhances lung cancer cell growth;promotes the angiogenesis;accelerates adipocyte differentiation;inhibits B cell development |
| hsa-mir-93 | ONCO | na |
| hsa-let-7a-1 | SUPP | Repress cell proliferation and growth;inhibits cell cycle progression; let-7f promotes angiogenesis;apoptotic signalin;induces both cell cycle arrest and cell death;contributes to epithelial immune responses against C. parvum infection |
| hsa-let-7a-2 | SUPP | Repress cell proliferation and growth;inhibits cell cycle progression; let-7f promotes angiogenesis;apoptotic signalin;induces both cell cycle arrest and cell death;contributes to epithelial immune responses against C. parvum infection |
| hsa-let-7a-3 | SUPP | Repress cell proliferation and growth;inhibits cell cycle progression; let-7f promotes angiogenesis;apoptotic signalin;induces both cell cycle arrest and cell death;contributes to epithelial immune responses against C. parvum infection |
| hsa-let-7b | SUPP | Repress cell proliferation and growth;inhibits cell cycle progression; let-7f promotes angiogenesis;apoptotic signalin;induces both cell cycle arrest and cell death;contributes to epithelial immune responses against C. parvum infection |
| hsa-let-7c | SUPP | Repress cell proliferation and growth;inhibits cell cycle progression; let-7f promotes angiogenesis;apoptotic signalin;induces both cell cycle arrest and cell death;contributes to epithelial immune responses against C. parvum infection |
| hsa-let-7d | SUPP | Repress cell proliferation and growth;inhibits cell cycle progression; let-7f promotes angiogenesis;apoptotic signalin;induces both cell cycle arrest and cell death;contributes to epithelial immune responses against C. parvum infection |
| hsa-let-7e | SUPP | Repress cell proliferation and growth;inhibits cell cycle progression; let-7f promotes angiogenesis;apoptotic signalin;induces both cell cycle arrest and cell death;contributes to epithelial immune responses against C. parvum infection |
| hsa-let-7f-1 | SUPP | Repress cell proliferation and growth;inhibits cell cycle progression; let-7f promotes angiogenesis;apoptotic signalin;induces both cell cycle arrest and cell death;contributes to epithelial immune responses against C. parvum infection |
| hsa-let-7f-2 | SUPP | Repress cell proliferation and growth;inhibits cell cycle progression; let-7f promotes angiogenesis;apoptotic signalin;induces both cell cycle arrest and cell death;contributes to epithelial immune responses against C. parvum infection |
| hsa-let-7g | SUPP | Repress cell proliferation and growth;inhibits cell cycle progression; let-7f promotes angiogenesis;apoptotic signalin;induces both cell cycle arrest and cell death;contributes to epithelial immune responses against C. parvum infection |
| hsa-let-7i | SUPP | Repress cell proliferation and growth;inhibits cell cycle progression; let-7f promotes angiogenesis;apoptotic signalin;induces both cell cycle arrest and cell death;contributes to epithelial immune responses against C. parvum infection |
| hsa-mir-124-1 | SUPP | the developing of neural tube; |
| hsa-mir-124-2 | SUPP | the developing of neural tube; |
| hsa-mir-124-3 | SUPP | the developing of neural tube; |
| hsa-mir-125a | SUPP | decreases cell growth |
| hsa-mir-125b-1 | SUPP | decreases cell growth |
| hsa-mir-125b-2 | SUPP | decreases cell growth |
| hsa-mir-127 | SUPP | is methylated in tumor cells; |
| hsa-mir-143 | SUPP | adipocyte differentiation |
| hsa-mir-145 | SUPP | Altered expression of miR-145 is related to clinicopathologic features of colorectal cancer |
| hsa-mir-15a | SUPP | Induce apoptosis;regulates cell cycle;controls cell cycle progression |
| hsa-mir-16-1 | SUPP | Induce apoptosis;regulates cell cycle;controls cell cycle progression |
| hsa-mir-181a-1 | SUPP | regulates B-cell and T-cell development;modulates T-cell sensitivity to antigens |
| hsa-mir-181a-2 | SUPP | regulates B-cell and T-cell development;modulates T-cell sensitivity to antigens |
| hsa-mir-181b-1 | SUPP | regulates B-cell and T-cell development;modulates T-cell sensitivity to antigens |
| hsa-mir-181b-2 | SUPP | regulates B-cell and T-cell development;modulates T-cell sensitivity to antigens |
| hsa-mir-181c | SUPP | regulates B-cell and T-cell development;modulates T-cell sensitivity to antigens |
| hsa-mir-195 | SUPP | na |
| hsa-mir-26a-1 | SUPP | cell growth inhibition; |
| hsa-mir-26a-2 | SUPP | cell growth inhibition; |
| hsa-mir-26b | SUPP | cell growth inhibition; |
| hsa-mir-29a | SUPP | regulates apoptosis |
| hsa-mir-29c | SUPP | regulates apoptosis |
| hsa-mir-34a | SUPP | mediate apopotosis; mediate induction of apoptosis, cell cycle arrest; promotes apoptosis;control of cell proliferation |
| hsa-mir-34b | SUPP | mediate apopotosis; mediate induction of apoptosis, cell cycle arrest; promotes apoptosis;control of cell proliferation |
| hsa-mir-34c | SUPP | mediate apopotosis; mediate induction of apoptosis, cell cycle arrest; promotes apoptosis;control of cell proliferation |
| hsa-mir-98 | SUPP | High mobility group A2 is a target for miRNA-98 in head and neck squamous cell carcinoma |

**Table 2. microRNA oncogenes and tumor suppressors used as the testing dataset in the prediction model** and their functions

| miRNA | Type | Function |
| --- | --- | --- |
| hsa-mir-150 | ONCO | Putative targets of this endogenous miRNAs included genes encoding death receptors, caspases, and other apoptosis-related genes;controls B cell differentiation; Inhibition of mir-150 decreases cell growth; |
| hsa-mir-18b | ONCO | miR-18 negatively regulates the anti-angiogenic connective tissue growth factor (CTGF); |
| hsa-mir-194-1 | ONCO | cell differentiation; |
| hsa-mir-194-2 | ONCO | cell differentiation; |
| hsa-mir-196a-1 | ONCO | differentiation; |
| hsa-mir-196a-2 | ONCO | differentiation; |
| hsa-mir-203 | ONCO | promotes epidermal differentiation; repress tumor suppressors |
| hsa-mir-20b | ONCO | Kis2 ncRNA overexpression in mouse tumors results in miR-20b accumulation |
| hsa-mir-224 | ONCO | Profiling microRNA expression in hepatocellular carcinoma reveals microRNA-224 up-regulation and apoptosis inhibitor-5 as a microRNA-224-specific target |
| hsa-mir-27a | ONCO | oncogene; cell cycle |
| hsa-mir-101-1 | SUPP | autoimmunity |
| hsa-mir-101-2 | SUPP | autoimmunity |
| hsa-mir-1-1 | SUPP | heart development |
| hsa-mir-1-2 | SUPP | heart development |
| hsa-mir-122 | SUPP | inhibits HCV; |
| hsa-mir-126 | SUPP | suppressors; vascular inflammation; |
| hsa-mir-141 | SUPP | regulate epithelial to mesenchymal transition |
| hsa-mir-200a | SUPP | regulate epithelial to mesenchymal transition; |
| hsa-mir-200b | SUPP | regulate epithelial to mesenchymal transition; |
| hsa-mir-200c | SUPP | regulate epithelial to mesenchymal transition; |
| hsa-mir-451 | SUPP | na |

**Table 3. Training dataset used in the prediction model.**

| miRNA | Type | Conservation | Size of precursor miRNA | Size of mature miRNA | Expression level |
| --- | --- | --- | --- | --- | --- |
| hsa-mir-106a | ONCO | 2 | 81 | 23 | 5353 |
| hsa-mir-221 | ONCO | 2 | 110 | 23 | 16240 |
| hsa-mir-214 | ONCO | 2 | 110 | 22 | 16913 |
| hsa-mir-21 | ONCO | 2 | 72 | 22 | 280020 |
| hsa-mir-19b-2 | ONCO | 2 | 96 | 23 | 85611 |
| hsa-mir-19b-1 | ONCO | 2 | 87 | 23 | 85611 |
| hsa-mir-19a | ONCO | 2 | 82 | 23 | 43569 |
| hsa-mir-222 | ONCO | 2 | 110 | 21 | 47505 |
| hsa-mir-18a | ONCO | 2 | 71 | 23 | 941 |
| hsa-mir-210 | ONCO | 1 | 110 | 22 | 2761 |
| hsa-mir-106b | ONCO | 2 | 82 | 21 | 11115 |
| hsa-mir-107 | ONCO | 2 | 81 | 23 | 496 |
| hsa-mir-17 | ONCO | 2 | 84 | 23 | 3462 |
| hsa-mir-155 | ONCO | 2 | 65 | 23 | 2428 |
| hsa-mir-146b | ONCO | 2 | 73 | 22 | 18411 |
| hsa-mir-146a | ONCO | 2 | 99 | 22 | 11133 |
| hsa-mir-191 | ONCO | 2 | 92 | 23 | 19532 |
| hsa-mir-24-1 | ONCO | 2 | 68 | 22 | 217882 |
| hsa-mir-24-2 | ONCO | 2 | 73 | 22 | 217882 |
| hsa-mir-93 | ONCO | 2 | 80 | 23 | 20951 |
| hsa-mir-92a-2 | ONCO | 1 | 75 | 22 | 96338 |
| hsa-mir-92a-1 | ONCO | 1 | 78 | 22 | 96338 |
| hsa-mir-373 | ONCO | 4 | 69 | 23 | 121 |
| hsa-mir-223 | ONCO | 2 | 110 | 22 | 50829 |
| hsa-mir-372 | ONCO | 3 | 67 | 23 | 332 |
| hsa-mir-20a | ONCO | 2 | 71 | 23 | 98995 |
| hsa-let-7i | SUPP | 1 | 84 | 22 | 22486 |
| hsa-mir-125b-1 | SUPP | 1 | 88 | 22 | 326604 |
| hsa-mir-125a | SUPP | 1 | 86 | 24 | 86533 |
| hsa-mir-124-3 | SUPP | 1 | 87 | 20 | 2662 |
| hsa-mir-124-2 | SUPP | 1 | 109 | 20 | 2662 |
| hsa-mir-124-1 | SUPP | 1 | 85 | 20 | 2662 |
| hsa-let-7a-3 | SUPP | 1 | 74 | 22 | 71294 |
| hsa-let-7b | SUPP | 1 | 83 | 22 | 53010 |
| hsa-let-7c | SUPP | 1 | 84 | 22 | 193761 |
| hsa-let-7g | SUPP | 1 | 84 | 22 | 105034 |
| hsa-let-7d | SUPP | 1 | 87 | 22 | 7886 |
| hsa-let-7e | SUPP | 1 | 79 | 22 | 1651 |
| hsa-let-7f-1 | SUPP | 1 | 87 | 22 | 87944 |
| hsa-mir-125b-2 | SUPP | 1 | 89 | 22 | 326604 |
| hsa-let-7f-2 | SUPP | 1 | 83 | 22 | 87944 |
| hsa-mir-181b-2 | SUPP | 2 | 89 | 23 | 338661 |
| hsa-let-7a-2 | SUPP | 1 | 72 | 22 | 71294 |
| hsa-mir-181c | SUPP | 2 | 110 | 22 | 533 |
| hsa-mir-34c | SUPP | 1 | 77 | 23 | 1402 |
| hsa-mir-34b | SUPP | 1 | 84 | 22 | 450 |
| hsa-mir-34a | SUPP | 1 | 110 | 22 | 6202 |
| hsa-mir-29c | SUPP | 1 | 88 | 22 | 41707 |
| hsa-mir-29a | SUPP | 1 | 64 | 22 | 110818 |
| hsa-mir-26b | SUPP | 2 | 77 | 21 | 135113 |
| hsa-mir-26a-2 | SUPP | 2 | 84 | 22 | 597908 |
| hsa-mir-181a-2 | SUPP | 2 | 110 | 23 | 14148 |
| hsa-mir-195 | SUPP | 2 | 87 | 21 | 86540 |
| hsa-mir-127 | SUPP | 3 | 97 | 22 | 5532 |
| hsa-mir-98 | SUPP | 1 | 119 | 22 | 1223 |
| hsa-mir-181b-1 | SUPP | 2 | 110 | 23 | 338661 |
| hsa-let-7a-1 | SUPP | 1 | 80 | 22 | 71294 |
| hsa-mir-181a-1 | SUPP | 2 | 110 | 23 | 14148 |
| hsa-mir-16-1 | SUPP | 2 | 89 | 22 | 232132 |
| hsa-mir-15a | SUPP | 2 | 83 | 22 | 28792 |
| hsa-mir-145 | SUPP | 2 | 88 | 23 | 43500 |
| hsa-mir-143 | SUPP | 2 | 106 | 21 | 14639 |
| hsa-mir-26a-1 | SUPP | 2 | 77 | 22 | 597908 |

**Table 4. Testing dataset used in the prediction model.**

| miRNA | Type | Conservation | Size of precursor miRNA | Size of mature miRNA | Expression level |
| --- | --- | --- | --- | --- | --- |
| hsa-mir-150 | ONCO | 3 | 79 | 21 | 58646 |
| hsa-mir-18b | ONCO | 2 | 71 | 22 | 0 |
| hsa-mir-194-1 | ONCO | 2 | 85 | 22 | 51345 |
| hsa-mir-194-2 | ONCO | 2 | 85 | 22 | 51345 |
| hsa-mir-196a-1 | ONCO | 2 | 85 | 22 | 4241 |
| hsa-mir-196a-2 | ONCO | 2 | 95 | 22 | 4241 |
| hsa-mir-203 | ONCO | 2 | 87 | 23 | 5763 |
| hsa-mir-20b | ONCO | 2 | 84 | 22 | 1879 |
| hsa-mir-224 | ONCO | 3 | 71 | 23 | 1753 |
| hsa-mir-27a | ONCO | 2 | 85 | 22 | 37665 |
| hsa-mir-101-1 | SUPP | 2 | 70 | 22 | 1950 |
| hsa-mir-101-2 | SUPP | 2 | 110 | 22 | 1950 |
| hsa-mir-1-1 | SUPP | 1 | 90 | 22 | 178790 |
| hsa-mir-1-2 | SUPP | 1 | 95 | 22 | 178790 |
| hsa-mir-122 | SUPP | 2 | 68 | 23 | 6370 |
| hsa-mir-126 | SUPP | 1 | 110 | 22 | 147365 |
| hsa-mir-141 | SUPP | 1 | 69 | 23 | 8136 |
| hsa-mir-200a | SUPP | 1 | 81 | 21 | 5724 |
| hsa-mir-200b | SUPP | 1 | 84 | 22 | 21126 |
| hsa-mir-200c | SUPP | 1 | 78 | 21 | 65078 |
| hsa-mir-451 | SUPP | 2 | 72 | 22 | 9071 |

**Table 5. Prediction results of the prediction model. Three false predictions were highlighted.**

| miRNA | Original Type | Prediction Type |
| --- | --- | --- |
| hsa-mir-150 | ONCO | ONCO |
| hsa-mir-18b | ONCO | ONCO |
| hsa-mir-194-1 | ONCO | ONCO |
| hsa-mir-194-2 | ONCO | ONCO |
| hsa-mir-196a-1 | ONCO | ONCO |
| hsa-mir-196a-2 | ONCO | ONCO |
| hsa-mir-203 | ONCO | ONCO |
| hsa-mir-20b | ONCO | ONCO |
| hsa-mir-224 | ONCO | ONCO |
| hsa-mir-27a | ONCO | ONCO |
| **hsa-mir-101-1** | **SUPP** | **ONCO** |
| hsa-mir-101-2 | SUPP | SUPP |
| hsa-mir-1-1 | SUPP | SUPP |
| hsa-mir-1-2 | SUPP | SUPP |
| **hsa-mir-122** | **SUPP** | **ONCO** |
| hsa-mir-126 | SUPP | SUPP |
| hsa-mir-141 | SUPP | SUPP |
| hsa-mir-200a | SUPP | SUPP |
| hsa-mir-200b | SUPP | SUPP |
| hsa-mir-200c | SUPP | SUPP |
| **hsa-mir-451** | **SUPP** | **ONCO** |
