# Supplementary material for: Human MicroRNA Oncogenes and Tumor Suppressors Show Significantly Different Biological Patterns: From Functions to Targets
Source: PLoS One. 2010 Sep 30;5(9):e13067. doi: 10.1371/journal.pone.0013067 (PMC2948010; doi:10.1371/journal.pone.0013067)
Supplement: File S2 — Cross-species conservation of human miRNAs. (0.69 MB DOC) [file pone.0013067.s005.doc]

**Supplementary File 2. Cross-species conservation of human miRNAs.**

| miRNA | Conservation Group |
| --- | --- |
| hsa-mir-1180 | 5 |
| hsa-mir-1200 | 5 |
| hsa-mir-1228 | 5 |
| hsa-mir-1229 | 5 |
| hsa-mir-1231 | 5 |
| hsa-mir-1238 | 5 |
| hsa-mir-1243 | 5 |
| hsa-mir-1252 | 5 |
| hsa-mir-1257 | 5 |
| hsa-mir-1260 | 5 |
| hsa-mir-1261 | 5 |
| hsa-mir-1268 | 5 |
| hsa-mir-1269 | 5 |
| hsa-mir-1270 | 5 |
| hsa-mir-1279 | 5 |
| hsa-mir-1304 | 5 |
| hsa-mir-1305 | 5 |
| hsa-mir-1308 | 5 |
| hsa-mir-1321 | 5 |
| hsa-mir-1468 | 5 |
| hsa-mir-1469 | 5 |
| hsa-mir-1470 | 5 |
| hsa-mir-1471 | 5 |
| hsa-mir-1537 | 5 |
| hsa-mir-1538 | 5 |
| hsa-mir-1539 | 5 |
| hsa-mir-1826 | 5 |
| hsa-mir-1908 | 5 |
| hsa-mir-1909 | 5 |
| hsa-mir-1910 | 5 |
| hsa-mir-1911 | 5 |
| hsa-mir-1912 | 5 |
| hsa-mir-1913 | 5 |
| hsa-mir-1914 | 5 |
| hsa-mir-1915 | 5 |
| hsa-mir-1972 | 5 |
| hsa-mir-1973 | 5 |
| hsa-mir-1974 | 5 |
| hsa-mir-1975 | 5 |
| hsa-mir-1976 | 5 |
| hsa-mir-1977 | 5 |
| hsa-mir-1978 | 5 |
| hsa-mir-1979 | 5 |
| hsa-mir-2052 | 5 |
| hsa-mir-2053 | 5 |
| hsa-mir-2054 | 5 |
| hsa-mir-2110 | 5 |
| hsa-mir-2113 | 5 |
| hsa-mir-220c | 5 |
| hsa-mir-548o | 5 |
| hsa-mir-571 | 5 |
| hsa-mir-585 | 5 |
| hsa-mir-596 | 5 |
| hsa-mir-602 | 5 |
| hsa-mir-606 | 5 |
| hsa-mir-608 | 5 |
| hsa-mir-620 | 5 |
| hsa-mir-623 | 5 |
| hsa-mir-629 | 5 |
| hsa-mir-647 | 5 |
| hsa-mir-659 | 5 |
| hsa-mir-921 | 5 |
| hsa-mir-17 | 2 |
| hsa-mir-18a | 2 |
| hsa-mir-20a | 2 |
| hsa-mir-93 | 2 |
| hsa-mir-106a | 2 |
| hsa-mir-106b | 2 |
| hsa-mir-18b | 2 |
| hsa-mir-20b | 2 |
| hsa-let-7a-1 | 1 |
| hsa-let-7a-2 | 1 |
| hsa-let-7a-3 | 1 |
| hsa-let-7b | 1 |
| hsa-let-7c | 1 |
| hsa-let-7d | 1 |
| hsa-let-7e | 1 |
| hsa-let-7f-1 | 1 |
| hsa-let-7f-2 | 1 |
| hsa-mir-98 | 1 |
| hsa-let-7g | 1 |
| hsa-let-7i | 1 |
| hsa-mir-30a | 2 |
| hsa-mir-30c-2 | 2 |
| hsa-mir-30d | 2 |
| hsa-mir-30b | 2 |
| hsa-mir-30c-1 | 2 |
| hsa-mir-30e | 2 |
| hsa-mir-15a | 2 |
| hsa-mir-16-1 | 2 |
| hsa-mir-16-2 | 2 |
| hsa-mir-15b | 2 |
| hsa-mir-195 | 2 |
| hsa-mir-181a-2 | 2 |
| hsa-mir-181b-1 | 2 |
| hsa-mir-181c | 2 |
| hsa-mir-181a-1 | 2 |
| hsa-mir-181b-2 | 2 |
| hsa-mir-181d | 2 |
| hsa-mir-29a | 1 |
| hsa-mir-29b-1 | 1 |
| hsa-mir-29b-2 | 1 |
| hsa-mir-29c | 1 |
| hsa-mir-19a | 2 |
| hsa-mir-19b-1 | 2 |
| hsa-mir-19b-2 | 2 |
| hsa-mir-25 | 1 |
| hsa-mir-92a-1 | 1 |
| hsa-mir-92a-2 | 1 |
| hsa-mir-92b | 1 |
| hsa-mir-9-1 | 1 |
| hsa-mir-9-2 | 1 |
| hsa-mir-9-3 | 1 |
| hsa-mir-125b-1 | 1 |
| hsa-mir-125a | 1 |
| hsa-mir-125b-2 | 1 |
| hsa-mir-154 | 3 |
| hsa-mir-369 | 3 |
| hsa-mir-377 | 3 |
| hsa-mir-381 | 3 |
| hsa-mir-382 | 3 |
| hsa-mir-323 | 3 |
| hsa-mir-453 | 3 |
| hsa-mir-409 | 3 |
| hsa-mir-410 | 3 |
| hsa-mir-487a | 3 |
| hsa-mir-494 | 3 |
| hsa-mir-496 | 3 |
| hsa-mir-539 | 3 |
| hsa-mir-487b | 3 |
| hsa-mir-655 | 3 |
| hsa-mir-656 | 3 |
| hsa-mir-1185-2 | 3 |
| hsa-mir-1185-1 | 3 |
| hsa-mir-300 | 3 |
| hsa-mir-200b | 1 |
| hsa-mir-141 | 1 |
| hsa-mir-200c | 1 |
| hsa-mir-200a | 1 |
| hsa-mir-429 | 1 |
| hsa-mir-520e | 4 |
| hsa-mir-515-1 | 4 |
| hsa-mir-519e | 4 |
| hsa-mir-520f | 4 |
| hsa-mir-515-2 | 4 |
| hsa-mir-519c | 4 |
| hsa-mir-520a | 4 |
| hsa-mir-526b | 4 |
| hsa-mir-519b | 4 |
| hsa-mir-525 | 4 |
| hsa-mir-523 | 4 |
| hsa-mir-518f | 4 |
| hsa-mir-520b | 4 |
| hsa-mir-518b | 4 |
| hsa-mir-526a-1 | 4 |
| hsa-mir-520c | 4 |
| hsa-mir-518c | 4 |
| hsa-mir-524 | 4 |
| hsa-mir-517a | 4 |
| hsa-mir-519d | 4 |
| hsa-mir-521-2 | 4 |
| hsa-mir-520d | 4 |
| hsa-mir-517b | 4 |
| hsa-mir-520g | 4 |
| hsa-mir-516b-2 | 4 |
| hsa-mir-526a-2 | 4 |
| hsa-mir-518e | 4 |
| hsa-mir-518a-1 | 4 |
| hsa-mir-518d | 4 |
| hsa-mir-516b-1 | 4 |
| hsa-mir-518a-2 | 4 |
| hsa-mir-517c | 4 |
| hsa-mir-520h | 4 |
| hsa-mir-521-1 | 4 |
| hsa-mir-522 | 4 |
| hsa-mir-519a-1 | 4 |
| hsa-mir-527 | 4 |
| hsa-mir-516a-1 | 4 |
| hsa-mir-516a-2 | 4 |
| hsa-mir-519a-2 | 4 |
| hsa-mir-1283-1 | 4 |
| hsa-mir-1283-2 | 4 |
| hsa-mir-124-1 | 1 |
| hsa-mir-124-2 | 1 |
| hsa-mir-124-3 | 1 |
| hsa-mir-7-1 | 1 |
| hsa-mir-7-2 | 1 |
| hsa-mir-7-3 | 1 |
| hsa-mir-103-2 | 2 |
| hsa-mir-103-1 | 2 |
| hsa-mir-107 | 2 |
| hsa-mir-99a | 1 |
| hsa-mir-100 | 1 |
| hsa-mir-99b | 1 |
| hsa-mir-218-1 | 2 |
| hsa-mir-218-2 | 2 |
| hsa-mir-23a | 2 |
| hsa-mir-23b | 2 |
| hsa-mir-135a-1 | 1 |
| hsa-mir-135a-2 | 1 |
| hsa-mir-135b | 1 |
| hsa-mir-133a-1 | 1 |
| hsa-mir-133a-2 | 1 |
| hsa-mir-133b | 1 |
| hsa-mir-196a-1 | 2 |
| hsa-mir-196a-2 | 2 |
| hsa-mir-196b | 2 |
| hsa-mir-10a | 1 |
| hsa-mir-10b | 1 |
| hsa-mir-130a | 2 |
| hsa-mir-301a | 2 |
| hsa-mir-130b | 2 |
| hsa-mir-301b | 2 |
| hsa-mir-27a | 2 |
| hsa-mir-27b | 2 |
| hsa-mir-1-2 | 1 |
| hsa-mir-206 | 1 |
| hsa-mir-1-1 | 1 |
| hsa-mir-34a | 1 |
| hsa-mir-34b | 1 |
| hsa-mir-34c | 1 |
| hsa-mir-199a-1 | 2 |
| hsa-mir-199a-2 | 2 |
| hsa-mir-199b | 2 |
| hsa-mir-24-1 | 2 |
| hsa-mir-24-2 | 2 |
| hsa-mir-204 | 2 |
| hsa-mir-211 | 2 |
| hsa-mir-26a-1 | 2 |
| hsa-mir-26b | 2 |
| hsa-mir-26a-2 | 2 |
| hsa-mir-219-1 | 1 |
| hsa-mir-219-2 | 1 |
| hsa-mir-101-1 | 2 |
| hsa-mir-101-2 | 2 |
| hsa-mir-128-1 | 2 |
| hsa-mir-128-2 | 2 |
| hsa-mir-153-1 | 1 |
| hsa-mir-153-2 | 1 |
| hsa-mir-221 | 2 |
| hsa-mir-222 | 2 |
| hsa-mir-22 | 2 |
| hsa-mir-216a | 1 |
| hsa-mir-216b | 1 |
| hsa-mir-194-1 | 2 |
| hsa-mir-194-2 | 2 |
| hsa-mir-148a | 2 |
| hsa-mir-152 | 2 |
| hsa-mir-148b | 2 |
| hsa-mir-28 | 3 |
| hsa-mir-151 | 3 |
| hsa-mir-205 | 2 |
| hsa-mir-184 | 1 |
| hsa-mir-21 | 2 |
| hsa-mir-365-1 | 2 |
| hsa-mir-365-2 | 2 |
| hsa-mir-214 | 2 |
| hsa-mir-192 | 2 |
| hsa-mir-215 | 2 |
| hsa-mir-31 | 1 |
| hsa-mir-212 | 2 |
| hsa-mir-132 | 2 |
| hsa-mir-183 | 1 |
| hsa-mir-223 | 2 |
| hsa-mir-371 | 3 |
| hsa-mir-372 | 3 |
| hsa-mir-32 | 2 |
| hsa-mir-33a | 1 |
| hsa-mir-33b | 1 |
| hsa-mir-302a | 2 |
| hsa-mir-302b | 2 |
| hsa-mir-302c | 2 |
| hsa-mir-302d | 2 |
| hsa-mir-302f | 2 |
| hsa-mir-96 | 1 |
| hsa-mir-129-1 | 2 |
| hsa-mir-129-2 | 2 |
| hsa-mir-105-1 | 3 |
| hsa-mir-105-2 | 3 |
| hsa-mir-138-2 | 2 |
| hsa-mir-138-1 | 2 |
| hsa-mir-190 | 1 |
| hsa-mir-190b | 1 |
| hsa-mir-217 | 1 |
| hsa-mir-187 | 2 |
| hsa-mir-145 | 2 |
| hsa-mir-127 | 3 |
| hsa-mir-193a | 2 |
| hsa-mir-193b | 2 |
| hsa-mir-142 | 2 |
| hsa-mir-140 | 2 |
| hsa-mir-210 | 1 |
| hsa-mir-224 | 3 |
| hsa-mir-198 | 4 |
| hsa-mir-376c | 3 |
| hsa-mir-376a-1 | 3 |
| hsa-mir-376b | 3 |
| hsa-mir-376a-2 | 3 |
| hsa-mir-144 | 2 |
| hsa-mir-143 | 2 |
| hsa-mir-122 | 2 |
| hsa-mir-338 | 2 |
| hsa-mir-95 | 3 |
| hsa-mir-545 | 3 |
| hsa-mir-421 | 3 |
| hsa-mir-1264 | 3 |
| hsa-mir-136 | 3 |
| hsa-mir-146a | 2 |
| hsa-mir-146b | 2 |
| hsa-mir-147 | 2 |
| hsa-mir-147b | 2 |
| hsa-mir-137 | 1 |
| hsa-mir-203 | 2 |
| hsa-mir-186 | 3 |
| hsa-mir-329-1 | 3 |
| hsa-mir-329-2 | 3 |
| hsa-mir-495 | 3 |
| hsa-mir-543 | 3 |
| hsa-mir-489 | 2 |
| hsa-mir-134 | 3 |
| hsa-mir-188 | 3 |
| hsa-mir-532 | 3 |
| hsa-mir-660 | 3 |
| hsa-mir-375 | 1 |
| hsa-mir-126 | 1 |
| hsa-mir-182 | 2 |
| hsa-mir-139 | 2 |
| hsa-mir-220a | 2 |
| hsa-mir-220b | 2 |
| hsa-mir-202 | 2 |
| hsa-mir-197 | 3 |
| hsa-mir-379 | 3 |
| hsa-mir-380 | 3 |
| hsa-mir-411 | 3 |
| hsa-mir-758 | 3 |
| hsa-mir-1197 | 3 |
| hsa-mir-450a-1 | 3 |
| hsa-mir-450a-2 | 3 |
| hsa-mir-450b | 3 |
| hsa-mir-455 | 2 |
| hsa-mir-511-1 | 3 |
| hsa-mir-511-2 | 3 |
| hsa-mir-512-1 | 3 |
| hsa-mir-512-2 | 3 |
| hsa-mir-513a-1 | 3 |
| hsa-mir-513a-2 | 3 |
| hsa-mir-506 | 3 |
| hsa-mir-507 | 3 |
| hsa-mir-508 | 3 |
| hsa-mir-509-1 | 3 |
| hsa-mir-510 | 3 |
| hsa-mir-514-1 | 3 |
| hsa-mir-514-2 | 3 |
| hsa-mir-514-3 | 3 |
| hsa-mir-509-2 | 3 |
| hsa-mir-509-3 | 3 |
| hsa-mir-513b | 3 |
| hsa-mir-513c | 3 |
| hsa-mir-449a | 2 |
| hsa-mir-449b | 2 |
| hsa-mir-383 | 2 |
| hsa-mir-363 | 2 |
| hsa-mir-500 | 3 |
| hsa-mir-501 | 3 |
| hsa-mir-502 | 3 |
| hsa-mir-431 | 3 |
| hsa-mir-326 | 3 |
| hsa-mir-325 | 3 |
| hsa-mir-451 | 2 |
| hsa-mir-448 | 3 |
| hsa-mir-155 | 2 |
| hsa-mir-296 | 3 |
| hsa-mir-367 | 2 |
| hsa-mir-320a | 3 |
| hsa-mir-320b-1 | 3 |
| hsa-mir-320c-1 | 3 |
| hsa-mir-320b-2 | 3 |
| hsa-mir-320d-1 | 3 |
| hsa-mir-320c-2 | 3 |
| hsa-mir-320d-2 | 3 |
| hsa-mir-424 | 3 |
| hsa-mir-324 | 3 |
| hsa-mir-370 | 3 |
| hsa-mir-378 | 3 |
| hsa-mir-361 | 3 |
| hsa-mir-499 | 2 |
| hsa-mir-454 | 2 |
| hsa-mir-433 | 3 |
| hsa-mir-208a | 2 |
| hsa-mir-208b | 2 |
| hsa-mir-483 | 3 |
| hsa-mir-503 | 3 |
| hsa-mir-542 | 3 |
| hsa-mir-299 | 3 |
| hsa-mir-346 | 3 |
| hsa-mir-345 | 3 |
| hsa-mir-342 | 3 |
| hsa-mir-340 | 3 |
| hsa-mir-412 | 3 |
| hsa-mir-339 | 3 |
| hsa-mir-191 | 2 |
| hsa-mir-337 | 3 |
| hsa-mir-335 | 3 |
| hsa-mir-150 | 3 |
| hsa-mir-331 | 3 |
| hsa-mir-330 | 3 |
| hsa-mir-485 | 3 |
| hsa-mir-185 | 3 |
| hsa-mir-328 | 3 |
| hsa-mir-297 | 3 |
| hsa-mir-298 | 3 |
| hsa-mir-362 | 3 |
| hsa-mir-432 | 3 |
| hsa-mir-541 | 3 |
| hsa-mir-505 | 3 |
| hsa-mir-484 | 3 |
| hsa-mir-486 | 3 |
| hsa-mir-490 | 2 |
| hsa-mir-493 | 3 |
| hsa-mir-497 | 3 |
| hsa-mir-425 | 2 |
| hsa-mir-149 | 3 |
| hsa-mir-452 | 3 |
| hsa-mir-374a | 3 |
| hsa-mir-374b | 3 |
| hsa-mir-384 | 3 |
| hsa-mir-664 | 3 |
| hsa-mir-1277 | 3 |
| hsa-mir-570 | 4 |
| hsa-mir-579 | 4 |
| hsa-mir-548a-1 | 4 |
| hsa-mir-548b | 4 |
| hsa-mir-548a-2 | 4 |
| hsa-mir-548a-3 | 4 |
| hsa-mir-603 | 4 |
| hsa-mir-548c | 4 |
| hsa-mir-548d-1 | 4 |
| hsa-mir-548d-2 | 4 |
| hsa-mir-548e | 4 |
| hsa-mir-548j | 4 |
| hsa-mir-548k | 4 |
| hsa-mir-548l | 4 |
| hsa-mir-548f-1 | 4 |
| hsa-mir-548f-2 | 4 |
| hsa-mir-548f-3 | 4 |
| hsa-mir-548f-4 | 4 |
| hsa-mir-548f-5 | 4 |
| hsa-mir-548g | 4 |
| hsa-mir-548n | 4 |
| hsa-mir-548m | 4 |
| hsa-mir-548h-1 | 4 |
| hsa-mir-548h-2 | 4 |
| hsa-mir-548h-3 | 4 |
| hsa-mir-548h-4 | 4 |
| hsa-mir-548p | 4 |
| hsa-mir-548i-1 | 4 |
| hsa-mir-548i-2 | 4 |
| hsa-mir-548i-3 | 4 |
| hsa-mir-548i-4 | 4 |
| hsa-mir-488 | 3 |
| hsa-mir-491 | 3 |
| hsa-mir-423 | 3 |
| hsa-mir-652 | 3 |
| hsa-mir-550-1 | 4 |
| hsa-mir-550-2 | 4 |
| hsa-mir-592 | 3 |
| hsa-mir-615 | 3 |
| hsa-mir-802 | 3 |
| hsa-mir-770 | 3 |
| hsa-mir-668 | 3 |
| hsa-mir-671 | 3 |
| hsa-mir-551a | 2 |
| hsa-mir-551b | 2 |
| hsa-mir-675 | 3 |
| hsa-mir-892a | 3 |
| hsa-mir-890 | 3 |
| hsa-mir-888 | 3 |
| hsa-mir-892b | 3 |
| hsa-mir-941-1 | 4 |
| hsa-mir-941-2 | 4 |
| hsa-mir-941-3 | 4 |
| hsa-mir-941-4 | 4 |
| hsa-mir-873 | 3 |
| hsa-mir-877 | 3 |
| hsa-mir-598 | 3 |
| hsa-mir-760 | 3 |
| hsa-mir-708 | 3 |
| hsa-mir-874 | 3 |
| hsa-mir-665 | 3 |
| hsa-mir-568 | 3 |
| hsa-mir-654 | 3 |
| hsa-mir-582 | 3 |
| hsa-mir-590 | 3 |
| hsa-mir-574 | 3 |
| hsa-mir-891a | 4 |
| hsa-mir-891b | 4 |
| hsa-mir-875 | 3 |
| hsa-mir-876 | 3 |
| hsa-mir-744 | 3 |
| hsa-mir-653 | 3 |
| hsa-mir-544 | 3 |
| hsa-mir-504 | 3 |
| hsa-mir-1224 | 3 |
| hsa-mir-1226 | 4 |
| hsa-mir-1225 | 3 |
| hsa-mir-1227 | 4 |
| hsa-mir-1302-1 | 4 |
| hsa-mir-1302-2 | 4 |
| hsa-mir-1302-3 | 4 |
| hsa-mir-1302-4 | 4 |
| hsa-mir-1302-5 | 4 |
| hsa-mir-1302-6 | 4 |
| hsa-mir-1302-7 | 4 |
| hsa-mir-1302-8 | 4 |
| hsa-mir-650 | 4 |
| hsa-mir-663 | 4 |
| hsa-mir-663b | 4 |
| hsa-mir-498 | 4 |
| hsa-mir-612 | 4 |
| hsa-mir-661 | 4 |
| hsa-mir-581 | 4 |
| hsa-mir-586 | 4 |
| hsa-mir-642 | 4 |
| hsa-mir-938 | 4 |
| hsa-mir-549 | 4 |
| hsa-mir-626 | 4 |
| hsa-mir-556 | 4 |
| hsa-mir-648 | 4 |
| hsa-mir-578 | 4 |
| hsa-mir-651 | 4 |
| hsa-mir-597 | 4 |
| hsa-mir-607 | 4 |
| hsa-mir-583 | 4 |
| hsa-mir-1271 | 3 |
| hsa-mir-600 | 4 |
| hsa-mir-638 | 4 |
| hsa-mir-553 | 4 |
| hsa-mir-643 | 4 |
| hsa-mir-939 | 4 |
| hsa-mir-662 | 4 |
| hsa-mir-576 | 4 |
| hsa-mir-640 | 4 |
| hsa-mir-920 | 4 |
| hsa-mir-589 | 3 |
| hsa-mir-657 | 4 |
| hsa-mir-492 | 4 |
| hsa-mir-373 | 4 |
| hsa-mir-552 | 4 |
| hsa-mir-599 | 3 |
| hsa-mir-593 | 4 |
| hsa-mir-933 | 4 |
| hsa-mir-1255a | 4 |
| hsa-mir-1255b-1 | 4 |
| hsa-mir-1255b-2 | 4 |
| hsa-mir-616 | 4 |
| hsa-mir-631 | 3 |
| hsa-mir-886 | 4 |
| hsa-mir-942 | 4 |
| hsa-mir-562 | 3 |
| hsa-mir-924 | 4 |
| hsa-mir-889 | 4 |
| hsa-mir-580 | 4 |
| hsa-mir-609 | 4 |
| hsa-mir-936 | 4 |
| hsa-mir-563 | 4 |
| hsa-mir-557 | 4 |
| hsa-mir-601 | 4 |
| hsa-mir-624 | 4 |
| hsa-mir-636 | 4 |
| hsa-mir-632 | 3 |
| hsa-mir-605 | 4 |
| hsa-mir-628 | 3 |
| hsa-mir-577 | 4 |
| hsa-mir-1306 | 2 |
| hsa-mir-885 | 3 |
| hsa-mir-584 | 3 |
| hsa-mir-625 | 4 |
| hsa-mir-569 | 4 |
| hsa-mir-922 | 4 |
| hsa-mir-572 | 4 |
| hsa-mir-558 | 4 |
| hsa-mir-611 | 4 |
| hsa-mir-940 | 3 |
| hsa-mir-944 | 4 |
| hsa-mir-934 | 4 |
| hsa-mir-765 | 4 |
| hsa-mir-633 | 4 |
| hsa-mir-649 | 4 |
| hsa-mir-422a | 4 |
| hsa-mir-639 | 4 |
| hsa-mir-627 | 4 |
| hsa-mir-554 | 4 |
| hsa-mir-767 | 3 |
| hsa-mir-618 | 4 |
| hsa-mir-887 | 4 |
| hsa-mir-567 | 4 |
| hsa-mir-644 | 4 |
| hsa-mir-619 | 4 |
| hsa-mir-1307 | 3 |
| hsa-mir-1285-1 | 4 |
| hsa-mir-1285-2 | 4 |
| hsa-mir-937 | 4 |
| hsa-mir-573 | 4 |
| hsa-mir-604 | 4 |
| hsa-mir-587 | 4 |
| hsa-mir-1300 | 3 |
| hsa-mir-1244 | 4 |
| hsa-mir-566 | 4 |
| hsa-mir-1233 | 4 |
| hsa-mir-1273 | 4 |
| hsa-mir-555 | 4 |
| hsa-mir-1297 | 4 |
| hsa-mir-1237 | 4 |
| hsa-mir-575 | 4 |
| hsa-mir-1282 | 3 |
| hsa-mir-1324 | 4 |
| hsa-mir-613 | 4 |
| hsa-mir-1278 | 4 |
| hsa-mir-1184 | 4 |
| hsa-mir-1234 | 4 |
| hsa-mir-1256 | 4 |
| hsa-mir-1262 | 4 |
| hsa-mir-1207 | 4 |
| hsa-mir-637 | 4 |
| hsa-mir-1298 | 3 |
| hsa-mir-1291 | 3 |
| hsa-mir-634 | 4 |
| hsa-mir-1178 | 4 |
| hsa-mir-1274a | 4 |
| hsa-mir-1274b | 4 |
| hsa-mir-1294 | 4 |
| hsa-mir-1284 | 3 |
| hsa-mir-646 | 4 |
| hsa-mir-935 | 3 |
| hsa-mir-1303 | 4 |
| hsa-mir-1205 | 4 |
| hsa-mir-1290 | 4 |
| hsa-mir-1253 | 4 |
| hsa-mir-564 | 4 |
| hsa-mir-1202 | 4 |
| hsa-mir-1254 | 4 |
| hsa-mir-1266 | 4 |
| hsa-mir-1323 | 4 |
| hsa-mir-1259 | 4 |
| hsa-mir-622 | 4 |
| hsa-mir-1245 | 4 |
| hsa-mir-1251 | 3 |
| hsa-mir-1248 | 3 |
| hsa-mir-561 | 4 |
| hsa-mir-1299 | 4 |
| hsa-mir-1289-1 | 4 |
| hsa-mir-1289-2 | 4 |
| hsa-mir-1246 | 4 |
| hsa-mir-1208 | 4 |
| hsa-mir-621 | 4 |
| hsa-mir-1179 | 3 |
| hsa-mir-1203 | 4 |
| hsa-mir-1206 | 4 |
| hsa-mir-1267 | 4 |
| hsa-mir-591 | 4 |
| hsa-mir-658 | 3 |
| hsa-mir-103-1-as | 4 |
| hsa-mir-103-2-as | 4 |
| hsa-mir-1272 | 4 |
| hsa-mir-1827 | 4 |
| hsa-mir-614 | 4 |
| hsa-mir-1280 | 4 |
| hsa-mir-1296 | 3 |
| hsa-mir-595 | 4 |
| hsa-mir-1276 | 4 |
| hsa-mir-630 | 4 |
| hsa-mir-559 | 4 |
| hsa-mir-645 | 4 |
| hsa-mir-302e | 4 |
| hsa-mir-1288 | 4 |
| hsa-mir-610 | 4 |
| hsa-mir-635 | 4 |
| hsa-mir-1322 | 4 |
| hsa-mir-720 | 4 |
| hsa-mir-1181 | 4 |
| hsa-mir-1249 | 3 |
| hsa-mir-1183 | 4 |
| hsa-mir-1247 | 4 |
| hsa-mir-1825 | 4 |
| hsa-mir-1204 | 4 |
| hsa-mir-617 | 4 |
| hsa-mir-1182 | 4 |
| hsa-mir-1275 | 4 |
| hsa-mir-1292 | 4 |
| hsa-mir-1295 | 4 |
| hsa-mir-766 | 4 |
| hsa-mir-641 | 4 |
| hsa-mir-588 | 4 |
| hsa-mir-1286 | 4 |
| hsa-mir-1258 | 4 |
| hsa-mir-1263 | 4 |
| hsa-mir-1265 | 4 |
| hsa-mir-1236 | 4 |
| hsa-mir-943 | 4 |
| hsa-mir-1293 | 4 |
| hsa-mir-1201 | 4 |
| hsa-mir-1281 | 3 |
| hsa-mir-1250 | 4 |
| hsa-mir-1287 | 3 |
| hsa-mir-769 | 3 |
| hsa-mir-1301 | 3 |
