# Supplementary material for: Human MicroRNA Oncogenes and Tumor Suppressors Show Significantly Different Biological Patterns: From Functions to Targets
Source: PLoS One. 2010 Sep 30;5(9):e13067. doi: 10.1371/journal.pone.0013067 (PMC2948010; doi:10.1371/journal.pone.0013067)
Supplement: File S3 — Distribution of SNPs in human miRNAs. (0.23 MB DOC) [file pone.0013067.s006.doc]

**Supplementary File 3. Distribution of SNPs in human miRNAs.**

| miRNA | SNP_ID | miRNA Type |
| --- | --- | --- |
| hsa-mir-92a-1 | rs9589207 | ONCO |
| hsa-mir-223 | rs34952329 | ONCO |
| hsa-mir-18a | rs41275866 | ONCO |
| hsa-mir-146a | rs61270459 | ONCO |
| hsa-mir-146a | rs2910164 | ONCO |
| hsa-mir-34a | rs35301225 | SUPP |
| hsa-mir-125a | rs12975333 | SUPP |
| hsa-mir-124-3 | rs34059726 | SUPP |
| hsa-mir-1302-2 | rs11266858 | Other |
| hsa-mir-1302-2 | rs4248191 | Other |
| hsa-mir-1302-2 | rs11266859 | Other |
| hsa-mir-1302-2 | rs422582 | Other |
| hsa-mir-1302-2 | rs422363 | Other |
| hsa-mir-92b | rs12759620 | Other |
| hsa-mir-558 | rs35999329 | Other |
| hsa-mir-559 | rs58450758 | Other |
| hsa-mir-217 | rs41291173 | Other |
| hsa-mir-216a | rs41291179 | Other |
| hsa-mir-1302-3 | rs2441622 | Other |
| hsa-mir-1302-3 | rs2441621 | Other |
| hsa-mir-1302-3 | rs7589328 | Other |
| hsa-mir-1302-3 | rs6542147 | Other |
| hsa-mir-663b | rs62165009 | Other |
| hsa-mir-1302-4 | rs57009980 | Other |
| hsa-mir-1244 | rs1804520 | Other |
| hsa-mir-149 | rs2292832 | Other |
| hsa-mir-564 | rs2292181 | Other |
| hsa-mir-1226 | rs59873154 | Other |
| hsa-mir-1324 | rs28620398 | Other |
| hsa-mir-1324 | rs7614638 | Other |
| hsa-mir-1324 | rs3008994 | Other |
| hsa-mir-1324 | rs10155043 | Other |
| hsa-mir-1324 | rs3008993 | Other |
| hsa-mir-1324 | rs58827088 | Other |
| hsa-mir-568 | rs28632138 | Other |
| hsa-mir-548i-1 | rs34864809 | Other |
| hsa-mir-570 | rs9860655 | Other |
| hsa-mir-943 | rs3034718 | Other |
| hsa-mir-943 | rs35401110 | Other |
| hsa-mir-943 | rs1077020 | Other |
| hsa-mir-1255b-1 | rs6841938 | Other |
| hsa-mir-1255a | rs28664200 | Other |
| hsa-mir-577 | rs34115976 | Other |
| hsa-mir-1274a | rs318039 | Other |
| hsa-mir-581 | rs788517 | Other |
| hsa-mir-581 | rs1694089 | Other |
| hsa-mir-581 | rs810917 | Other |
| hsa-mir-449b | rs10061133 | Other |
| hsa-mir-9-2 | rs41265488 | Other |
| hsa-mir-583 | rs10697860 | Other |
| hsa-mir-1244 | rs1804520 | Other |
| hsa-mir-1289-2 | rs35296450 | Other |
| hsa-mir-1289-2 | rs35731356 | Other |
| hsa-mir-1294 | rs13186787 | Other |
| hsa-mir-1303 | rs34889453 | Other |
| hsa-mir-1303 | rs33982250 | Other |
| hsa-mir-585 | rs62376934 | Other |
| hsa-mir-585 | rs62376935 | Other |
| hsa-mir-1229 | rs2291418 | Other |
| hsa-mir-548a-1 | rs12197631 | Other |
| hsa-mir-339 | rs13232101 | Other |
| hsa-mir-590 | rs6971711 | Other |
| hsa-mir-489 | rs35930643 | Other |
| hsa-mir-25 | rs41274221 | Other |
| hsa-mir-96 | rs41274239 | Other |
| hsa-mir-183 | rs41281222 | Other |
| hsa-mir-595 | rs4909237 | Other |
| hsa-mir-596 | rs61388742 | Other |
| hsa-mir-1322 | rs59878596 | Other |
| hsa-mir-486 | rs59908561 | Other |
| hsa-mir-1206 | rs2114358 | Other |
| hsa-mir-1208 | rs56863230 | Other |
| hsa-mir-1208 | rs2648841 | Other |
| hsa-mir-939 | rs35486628 | Other |
| hsa-mir-1234 | rs2291134 | Other |
| hsa-mir-1302-2 | rs11266858 | Other |
| hsa-mir-1302-2 | rs4248191 | Other |
| hsa-mir-1302-2 | rs11266859 | Other |
| hsa-mir-1302-2 | rs422582 | Other |
| hsa-mir-1302-2 | rs422363 | Other |
| hsa-mir-1299 | rs62555121 | Other |
| hsa-mir-1308 | rs7051072 | Other |
| hsa-mir-548f-5 | rs60180387 | Other |
| hsa-mir-532 | rs456615 | Other |
| hsa-mir-532 | rs456617 | Other |
| hsa-mir-1321 | rs36100620 | Other |
| hsa-mir-320d-2 | rs5907732 | Other |
| hsa-mir-888 | rs5965660 | Other |
| hsa-mir-891a | rs5965990 | Other |
| hsa-mir-513a-1 | rs35027589 | Other |
| hsa-mir-506 | rs5905008 | Other |
| hsa-mir-509-2 | rs36092315 | Other |
| hsa-mir-1184 | rs56191956 | Other |
| hsa-mir-1184 | rs56191956 | Other |
| hsa-mir-1184 | rs56191956 | Other |
| hsa-mir-1265 | rs11259096 | Other |
| hsa-mir-603 | rs11014002 | Other |
| hsa-mir-604 | rs2368393 | Other |
| hsa-mir-604 | rs2368392 | Other |
| hsa-mir-938 | rs12416605 | Other |
| hsa-mir-605 | rs2043556 | Other |
| hsa-mir-606 | rs34610391 | Other |
| hsa-mir-607 | rs12778876 | Other |
| hsa-mir-607 | rs12780546 | Other |
| hsa-mir-608 | rs58078477 | Other |
| hsa-mir-608 | rs4919510 | Other |
| hsa-mir-1307 | rs7911488 | Other |
| hsa-mir-202 | rs12355840 | Other |
| hsa-mir-1908 | rs174561 | Other |
| hsa-mir-194-2 | rs11231898 | Other |
| hsa-mir-612 | rs550894 | Other |
| hsa-mir-612 | rs12803915 | Other |
| hsa-mir-1304 | rs2155248 | Other |
| hsa-mir-548l | rs11020790 | Other |
| hsa-mir-548l | rs13447640 | Other |
| hsa-mir-100 | rs57448873 | Other |
| hsa-mir-141 | rs34385807 | Other |
| hsa-mir-1244 | rs1804520 | Other |
| hsa-mir-1244 | rs1804520 | Other |
| hsa-mir-196a-2 | rs11614913 | Other |
| hsa-mir-548c | rs17120527 | Other |
| hsa-mir-617 | rs12815353 | Other |
| hsa-mir-618 | rs2682818 | Other |
| hsa-mir-492 | rs2289030 | Other |
| hsa-mir-619 | rs34651680 | Other |
| hsa-mir-620 | rs10549054 | Other |
| hsa-mir-620 | rs5801168 | Other |
| hsa-mir-620 | rs3043743 | Other |
| hsa-mir-620 | rs34380284 | Other |
| hsa-mir-620 | rs34551929 | Other |
| hsa-mir-1178 | rs7311975 | Other |
| hsa-mir-622 | rs59274393 | Other |
| hsa-mir-208b | rs2754157 | Other |
| hsa-mir-624 | rs57264777 | Other |
| hsa-mir-625 | rs12894182 | Other |
| hsa-mir-1260 | rs28909969 | Other |
| hsa-mir-431 | rs12884005 | Other |
| hsa-mir-379 | rs61991156 | Other |
| hsa-mir-299 | rs41286566 | Other |
| hsa-mir-329-1 | rs34557733 | Other |
| hsa-mir-300 | rs12894467 | Other |
| hsa-mir-1185-2 | rs11844707 | Other |
| hsa-mir-453 | rs56103835 | Other |
| hsa-mir-154 | rs41286570 | Other |
| hsa-mir-412 | rs61992671 | Other |
| hsa-mir-656 | rs58834075 | Other |
| hsa-mir-1268 | rs28599926 | Other |
| hsa-mir-211 | rs34520022 | Other |
| hsa-mir-1233 | rs347881 | Other |
| hsa-mir-1233 | rs347882 | Other |
| hsa-mir-1233 | rs347881 | Other |
| hsa-mir-1233 | rs347882 | Other |
| hsa-mir-627 | rs2620381 | Other |
| hsa-mir-1282 | rs11269 | Other |
| hsa-mir-147b | rs56073218 | Other |
| hsa-mir-631 | rs5745925 | Other |
| hsa-mir-184 | rs41280052 | Other |
| hsa-mir-1276 | rs34381260 | Other |
| hsa-mir-7-2 | rs41276930 | Other |
| hsa-mir-1302-2 | rs422363 | Other |
| hsa-mir-1302-2 | rs422582 | Other |
| hsa-mir-1302-2 | rs11266859 | Other |
| hsa-mir-1302-2 | rs4248191 | Other |
| hsa-mir-1302-2 | rs11266858 | Other |
| hsa-mir-662 | rs9745376 | Other |
| hsa-mir-940 | rs35356504 | Other |
| hsa-mir-1826 | rs1987294 | Other |
| hsa-mir-1826 | rs62030476 | Other |
| hsa-mir-140 | rs7205289 | Other |
| hsa-mir-1253 | rs7217038 | Other |
| hsa-mir-548h-3 | rs9913045 | Other |
| hsa-mir-423 | rs61093106 | Other |
| hsa-mir-423 | rs6505162 | Other |
| hsa-mir-193a | rs60406007 | Other |
| hsa-mir-365-2 | rs35143473 | Other |
| hsa-mir-923 | rs4796042 | Other |
| hsa-mir-923 | rs62062408 | Other |
| hsa-mir-923 | rs12165042 | Other |
| hsa-mir-923 | rs28625679 | Other |
| hsa-mir-633 | rs17759989 | Other |
| hsa-mir-187 | rs41274312 | Other |
| hsa-mir-122 | rs41292412 | Other |
| hsa-mir-1302-2 | rs11266858 | Other |
| hsa-mir-1302-2 | rs4248191 | Other |
| hsa-mir-1302-2 | rs11266859 | Other |
| hsa-mir-1302-2 | rs422582 | Other |
| hsa-mir-1302-2 | rs422363 | Other |
| hsa-mir-220b | rs1053262 | Other |
| hsa-mir-1181 | rs2569788 | Other |
| hsa-mir-27a | rs895819 | Other |
| hsa-mir-27a | rs11671784 | Other |
| hsa-mir-639 | rs45556632 | Other |
| hsa-mir-639 | rs35149836 | Other |
| hsa-mir-1283-1 | rs57111412 | Other |
| hsa-mir-520c | rs7255628 | Other |
| hsa-mir-521-2 | rs13382089 | Other |
| hsa-mir-516b-2 | rs10670323 | Other |
| hsa-mir-516b-2 | rs33953969 | Other |
| hsa-mir-516b-2 | rs10583889 | Other |
| hsa-mir-518e | rs34416818 | Other |
| hsa-mir-518a-1 | rs61636451 | Other |
| hsa-mir-520h | rs56013413 | Other |
| hsa-mir-521-1 | rs2561251 | Other |
| hsa-mir-516a-1 | rs2569389 | Other |
| hsa-mir-1274b | rs7253945 | Other |
| hsa-mir-663 | rs28670321 | Other |
| hsa-mir-663 | rs2019798 | Other |
| hsa-mir-663 | rs7266947 | Other |
| hsa-mir-499 | rs3746444 | Other |
| hsa-mir-499 | rs7267163 | Other |
| hsa-mir-645 | rs35645123 | Other |
| hsa-mir-646 | rs6513496 | Other |
| hsa-mir-646 | rs6513497 | Other |
| hsa-mir-1-1 | rs6122014 | Other |
| hsa-mir-941-1 | rs56202554 | Other |
| hsa-mir-941-1 | rs7268785 | Other |
| hsa-mir-941-1 | rs2427556 | Other |
| hsa-mir-941-1 | rs55795631 | Other |
| hsa-mir-941-1 | rs6089780 | Other |
| hsa-mir-941-2 | rs34604519 | Other |
| hsa-mir-941-3 | rs12625445 | Other |
| hsa-mir-941-3 | rs35544770 | Other |
| hsa-mir-941-3 | rs12625454 | Other |
| hsa-mir-650 | rs11558654 | Other |
| hsa-mir-650 | rs5996397 | Other |
| hsa-mir-548j | rs4822739 | Other |
| hsa-mir-548j | rs12161068 | Other |
