# Supplementary material for: Human MicroRNA Oncogenes and Tumor Suppressors Show Significantly Different Biological Patterns: From Functions to Targets
Source: PLoS One. 2010 Sep 30;5(9):e13067. doi: 10.1371/journal.pone.0013067 (PMC2948010; doi:10.1371/journal.pone.0013067)
Supplement: File S4 — Distribution of miRNA oncogenes and tumor suppressors on chromosome regions associated with human cancer. "D" represents deleted regions in cancer and "A" represents amplified regions in cancer. (0.05 MB DOC) [file pone.0013067.s007.doc]

**Supplementary File 4.** **Distribution of miRNA oncogenes and tumor suppressors on chromosome regions associated with human cancer. “D” represents deleted regions in cancer and “A” represents amplified regions in cancer.**

| miRNA | miRNA Type | Genomic region |
| --- | --- | --- |
| mir-106a | ONCO | D |
| mir-155 | ONCO | A |
| mir-17 | ONCO | A |
| mir-18a | ONCO | A |
| mir-19a | ONCO | A |
| mir-19b-1 | ONCO | A |
| mir-19b-2 | ONCO | D |
| mir-20a | ONCO | A |
| mir-21 | ONCO | A |
| mir-210 | ONCO | D |
| mir-24-1 | ONCO | D |
| mir-92a-1 | ONCO | A |
| mir-92a-2 | ONCO | D |
| let-7a-1 | SUPP | D |
| let-7a-2 | SUPP | D |
| let-7c | SUPP | D |
| let-7d | SUPP | D |
| let-7f-1 | SUPP | D |
| let-7g | SUPP | D |
| let-7i | SUPP | A |
| mir-124-1 | SUPP | A |
| mir-125b-1 | SUPP | D |
| mir-125b-2 | SUPP | D |
| mir-127 | SUPP | D |
| mir-143 | SUPP | D |
| mir-145 | SUPP | D |
| mir-15a | SUPP | D |
| mir-16-1 | SUPP | D |
| mir-181a-1 | SUPP | D |
| mir-181a-2 | SUPP | D |
| mir-195 | SUPP | D |
| mir-26a-1 | SUPP | D |
| mir-26a-2 | SUPP | D |
| mir-29a | SUPP | D |
| mir-34a | SUPP | D |
| mir-34b | SUPP | D |
