# Supplementary material for: Human MicroRNA Oncogenes and Tumor Suppressors Show Significantly Different Biological Patterns: From Functions to Targets
Source: PLoS One. 2010 Sep 30;5(9):e13067. doi: 10.1371/journal.pone.0013067 (PMC2948010; doi:10.1371/journal.pone.0013067)
Supplement: File S5 — Supplementary results for "Human MicroRNA Oncogenes and Tumor Suppressors Show Significantly Different Biological Patterns: From Functions to Targets." (0.06 MB DOC) [file pone.0013067.s008.doc]

Supplementary File 5. Supplementary Results for “Human microRNA oncogenes and tumor suppressors show significantly different biological patterns: from functions to targets”

# Size of miRNAs

We found that miRNAs have different size (length) distributions both for precursor sequences and for mature sequences. For precursor miRNAs, t-miRNAs are a little shorter than o-miRNAs (P=0.15, Wilcoxon test) and p-oncomirs are shorter than p-mirsupps (P=0.03, Wilcoxon test). By contrast, a converse pattern was found for mature miRNAs. t-miRNAs are longer than o-miRNAs (P=0.002, Wilcoxon test) and p-oncomirs are longer than p-mirsupps (P=0.008, Wilcoxon test). This converse pattern for precursor miRNAs (o-miRNAs > p-mirsupps > p-oncomir) and mature miRNAs (o-miRNAs < p-mirsupps < p-oncomir) raises the possibility that there may be a negative correlation between the sizes of the precursor miRNAs and the mature miRNAs. Surprisingly, the correlation is positive (R=0.88, P=0.0005, Spearman’s correlation). This result suggests that different ratios of bases are removed from these three groups of miRNAs when their precursors are processed into mature sequences. The o-miRNAs remove the greatest number, followed by the p-mirsupps and the p-oncomirs. One possible reason for this difference in size is the material and energy cost during miRNA biogenesis. The more copies of a gene required by the organism, the greater the burden in terms of material and energy [1], and this principle can be used to explain why the precursor size of t-miRNA (with higher expression level) is shorter than that of o-miRNAs. However, this does not explain why the precursor p-suppmirs (higher expression level) are longer than precursor p-oncomirs nor explain why mature o-miRNAs (lower expression level) are shorter than the mature t-miRNAs. Because miRNAs are very small compared with protein-coding genes (number of bases- dozens vs. hundreds) and their absolute numbers are also very small (only 1,000 vs. 30,000 protein-coding genes), their material and energy requirements are likely not as large as protein-coding genes. Therefore, there must be other factors affecting miRNA size, and examination of the process of miRNA biogenesis may provide insights. miRNAs biogenesis is very complex, and many pathways can connect precursor miRNAs with mature miRNAs, and therefore much about miRNA biogenesis remains to be explored[2]. Some evidence for the roles of different miRNAs segments in miRNA biogenesis is emerging but data are still limited. For example, during the process of pre-miRNA cleavage by the Drosha–DGCR8 microprocessor complex, the loop region is not as critical as the double-stranded stem and the unpaired flanking regions for DGCR8 binding and Drosha cleavage[3,4,5]. We believe that as mechanisms of miRNA biogenesis are understood in greater detail, the reasons underlying different patterns of precursor miRNA and mature miRNA size which exist among p-oncomirs, p-mirsupps, and o-miRNAs will be found.

# Free energy of miRNA secondary structure

A stable secondary structure in a state of low free energy is necessary for a miRNA to play its role normally. In view of their importance, p-oncomirs and p-mirsupps may be more stable, and their secondary structures may therefore have lower free energy. Because longer RNA molecules normally have more paired bases, they normally have lower free energy. Therefore, it was necessary to first normalize the free energy of miRNAs by dividing their free energy by their lengths [6]. Carrying out this analysis, we did not find any significant differences in free energy among p-oncomirs, p-mirsupps, and o-miRNAs. Because of their conservation and fundamental functions, mutations occurring in p-oncomirs and p-mirsupps tend to be more deleterious. In energy terms, we hypothesize that these miRNAs tend to be in a lower free energy state than their mutants. In order to address this, we calculated the free energy for each mutant. As a result, we found that more mutants of t-miRNAs do in fact show increased free energy as compared with those of o-miRNAs (median percentage: 0.682 vs. 0.659, P=0.007, Wilcoxon test). However, no significant difference was found between p-oncomirs and p-mirsupps. Moreover, mutations occurring in the mature regions tend to be more deleterious. Indeed, for both t-miRNAs and o-miRNAs, mutations in mature regions more frequently increase the free energy (P=0.05 and P=0.007, respectively, paired t test). Of even gtreater interest, mutations in mature regions of t-miRNAs increase free energy more substantially than those of o-miRNAs (median percentage: 0.773 vs. 0.659, P=0.007, Wilcoxon test). No significant difference was found between p-oncomirs and p-mirsupps. These results suggest that from an energy standpoint, mutations in t-miRNAs may be more deleterious than those in o-miRNAs, and mutations in mature regions may be more deleterious than those in other regions. Although no significant differences were found between p-oncomirs and p-mirsupps, it is still useful to note that these findings are of potential value in the evaluation of phenotypic consequences of miRNA mutations.

# Transcription factors that regulate p-oncomirs and p-mirsupps

The transcription of miRNA genes is activated or repressed by transcription factors (TFs). Therefore, it is reasonable to speculate that the p-oncomirs are preferentially activated by TF oncogenes or repressed by TF suppressors, and the p-mirsupps are preferentially activated by TF suppressors or repressed by TF oncogenes. This type of regulation represents a balanced TF-miRNA relationship. We obtained the regulatory relationships between TF and miRNAs from TransmiR [7], and found that 85.8% (91/106) of the TF-miRNA relationships are balanced.

# Prediction of novel p-oncomirs and p-mirsupps

To confirm the ability of uncovered patterns to discriminate novel p-oncomirs (assigned as positive samples here) and p-mirsupps (assigned as negative samples here), here, we used four biological characteristics that are available for many miRNAs: evolutionary conservation, size of precursor miRNA, size of mature miRNA, and expression profile to establish a support vector machine (SVM) based classifier to discriminate p-oncomirs from p-mirsupps. In this study a SVM package, LIBSVM [8], was used. In SVM, the Radial basis function (RBF) was used as the kernel function. We trained the classifier using the samples used in the analysis. We first evaluate the performance of this classifier with 10-fold cross-validation. The result showed an accuracy of 79.4%, a sensitivity of 73.1%, and a specificity of 83.8%. We then applied the trained classifier to the novel independent miRNAs culled from literature published from June 2008 to April 2009. As a result, the classifier achieved a prediction accuracy of 85.7%, a sensitivity of 100%, and a specificity of 72.7%. (Supplementary File1). This degree of accuracy was achieved by using only four features. A higher degree of accuracy would be expected if other features such as targets and transcription factors were available.

# References

1. Eisenberg E, Levanon EY (2003) Human housekeeping genes are compact. Trends Genet 19: 362-365.

2. Winter J, Jung S, Keller S, Gregory RI, Diederichs S (2009) Many roads to maturity: microRNA biogenesis pathways and their regulation. Nat Cell Biol 11: 228-234.

3. Han J, Lee Y, Yeom KH, Nam JW, Heo I, et al. (2006) Molecular basis for the recognition of primary microRNAs by the Drosha-DGCR8 complex. Cell 125: 887-901.

4. Zeng Y, Cullen BR (2003) Sequence requirements for micro RNA processing and function in human cells. RNA 9: 112-123.

5. Zeng Y, Cullen BR (2005) Efficient processing of primary microRNA hairpins by Drosha requires flanking nonstructured RNA sequences. J Biol Chem 280: 27595-27603.

6. Pervouchine DD, Graber JH, Kasif S (2003) On the normalization of RNA equilibrium free energy to the length of the sequence. Nucleic Acids Res 31: e49.

7. Wang J, Lu M, Qiu C, Cui Q (2010) TransmiR: a transcription factor-microRNA regulation database. Nucleic Acids Res 38: D119-122.

8. Chang CC, Lin, C.J. (2001) LIBSVM : a library for support vector machines.
